# Charge density view on bicalutamide molecular interactions in the monoclinic polymorph and androgen receptor binding pocket 

**DOI:** 10.1107/S2052252519014416

**Published:** 2020-01-01

**Authors:** Alexander A. Korlyukov, Maura Malinska, Anna V. Vologzhanina, Mikhail S. Goizman, Damian Trzybinski, Krzysztof Wozniak

**Affiliations:** a A. N. Nesmeyanov Institute of Organoelement Compounds of Russian Academy of Sciences, Vavilov St. 28, Moscow 119991, Russian Federation; b Biological and Chemical Research Centre, Department of Chemistry, University of Warsaw, Żwirki i Wigury 101, Warszawa 02089, Poland; c Drug Technology Co, 2a Rabochaya Street, Chimki, Moscow Oblast 141400, Russian Federation

**Keywords:** structure determination, drug discovery, protein structures, X-ray crystallography, intermolecular interactions

## Abstract

High-resolution single-crystal X-ray measurements of the monoclinic polymorph of bicalutamide and the aspherical atom databank approach have served as a basis for a reconstruction of the charge density distribution of the drug and its androgen receptor (AR) and albumin complexes. The contributions of various types of intermolecular interactions to the total crystal energy or ligand:AR energy were estimated. The cyan and amide groups secured the ligand placement in the albumin (Lys-137) and the AR binding pocket (Leu-704, Asn-705, Arg-752), and also determined the packing of the small-molecule crystals. The total electrostatic interaction energy on average was −230 kJ mol^−1^, comparable with the electrostatic lattice energy of the monoclinic bicalutamide polymorph. This is the result of similar distributions of electropositive and electronegative regions on the experimental and theoretical molecular electrostatic potential maps despite differences in molecular conformations. In general, bicalutamide interacted with the studied proteins with similar electrostatic interaction energies and adjusted its conformation and electrostatic potential to fit the binding pocket in such a way as to enhance the interactions, *e.g.* hydrogen bonds and π⋯π stacking.

## Abstract 2

Experimental and theoretical electron density distributions and the nature of intermolecular interactions of bicalutamide in its monoclinic polymorph and in androgen receptor complexes are reported.

## 1. Introduction   

One of the most challenging problems in molecular biology is the search for the mechanism of the agonistic or antagonistic activities of genes related to the growth of tumour tissue. For example, in prostate cancer, many tumours are hormone-dependent, meaning that drugs that can block or inhibit androgen receptors might have potential as chemotherapies. These potential drugs can be divided into two groups: steroidal (those that contain steroid fragments) and non-steroidal. Herein, we present the results of charge density studies of the compound most commonly used to treat prostate cancer: racemic monoclinic bicalutamide (**Bic**), commercially available as *Casodex* (Scheme 1). **Bic** is a non-steroidal drug that possesses low solubility in water (5 mg l^−1^, according to https://www.drugbank.ca/drugs/DB01128) and demonstrates antiandrogen activity and is a selective antagonist of the androgen receptor (AR). Antiandrogens are AR ligands that antagonize the actions of androgens by competing for AR binding sites. Antiandrogens can be both steroidal and non-steroidal drugs. Toluidide derivatives such as **Bic** are antiandrogens without themselves having androgenic properties; this lack of androgenic properties makes them suitable for use in the treatment of prostate cancer (Tan *et al.*, 2012 ▸). Furthermore, **Bic** is on the World Health Organization’s List of Essential Medicines. 
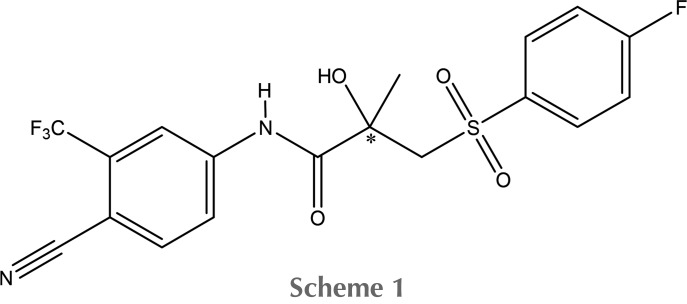


**Figure 1:**
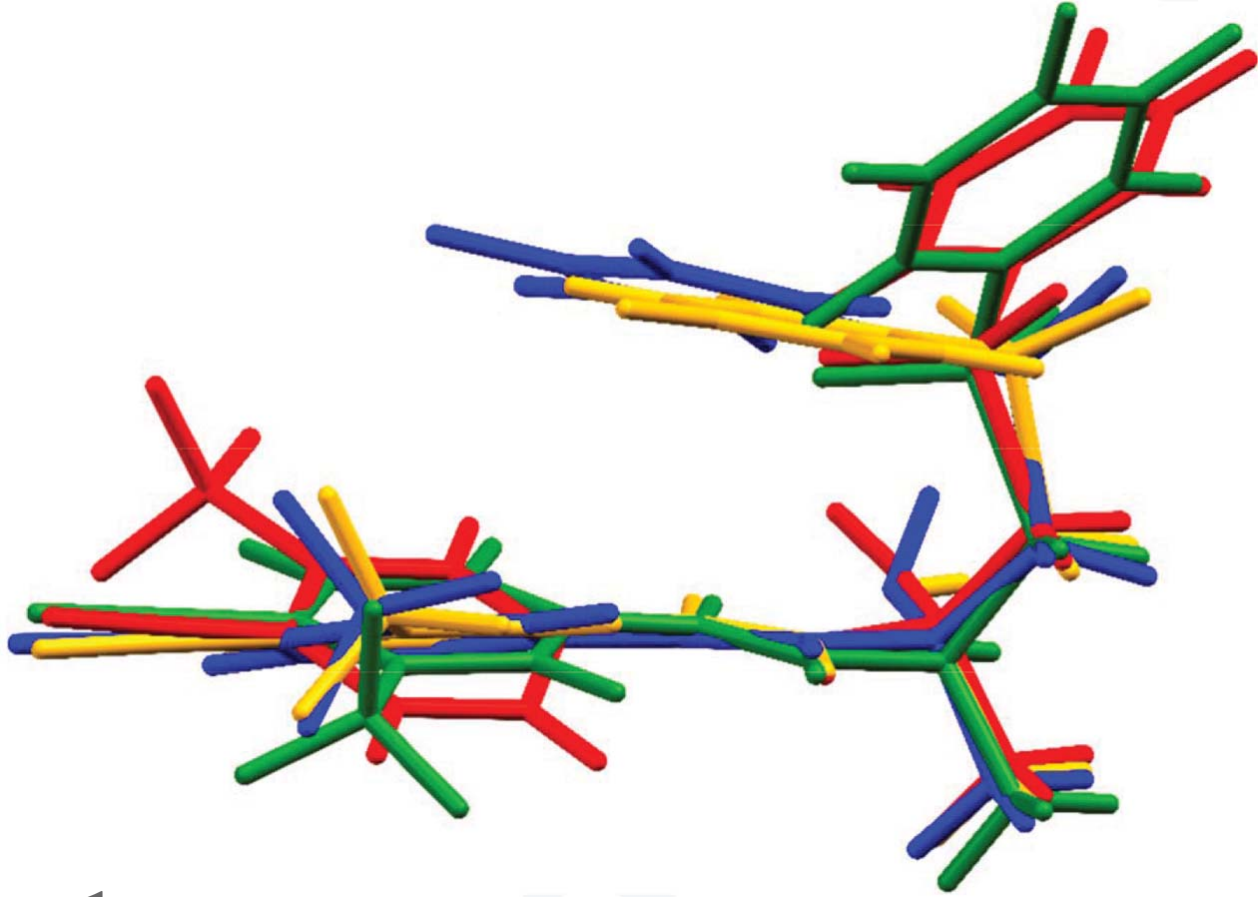
Overlay of the **Bic** moieties in the two polymorphs (monoclinic, red; triclinic, orange), in the AR binding pocket (green) and in the albumin binding pocket (blue). Hydrogen atoms have been omitted for clarity. Superimposed atoms are the chiral carbon atoms and their four neighbours.

Crystal structures of two polymorphs [triclinic and monoclinic (Bis *et al.*, 2007 ▸; Vega *et al.*, 2007 ▸)], several cocrystals (Surov *et al.*, 2016 ▸) and the complexes of **Bic** with various receptors were studied using X-ray diffraction (Hsu *et al.*, 2014 ▸; Bohl *et al.*, 2005 ▸; Osguthorpe & Hagler, 2011 ▸; Mast *et al.*, 2013 ▸), quantum chemical calculations (Bonomo *et al.*, 2016 ▸; Le *et al.*, 2009 ▸) and molecular dynamics (MD) simulations (Hsu *et al.*, 2014 ▸). Comparison of **Bic** conformations derived from XRD data taken from the Cambridge Structural Database (CSD) and the Protein Databank (PDB) are visualized in Fig. 1 ▸ as superimposed chiral atoms. The local neighbourhood of these atoms undoubtedly indicates the inherent flexibility of the molecule. Sulfur-containing bonds were found to be the most flexible, followed by C—N(H)—C_Ar_ bonds. The same level of conformational flexibility was obtained from *ab initio* calculations of **Bic** (Dhaked *et al.*, 2012 ▸). Note also that none of the polymorphs of **Bic** represent conformations that have been found in complexes with macromolecules, but the triclinic polymorph and co-crystals tend to form a π⋯π stacking arrangement of two aryl rings such as that found in the complex of **Bic** with the heme molecule of human CYP46A1 P450 (Mast *et al.*, 2013 ▸) and albumin.

Several types of ligand–receptor complexes are present in the PDB, but the nature and the strength of intermolecular bonds present differ significantly. At first glance, the hydroxyl group, which can form strong hydrogen bonds, plays the most significant role in bonding between the receptor and the peptide chains. XRD analysis also indicated that the nitrogen atom of the C≡N group (Mast *et al.*, 2013 ▸) can coordinate to the iron atom of cytochrome. In the absence of the heme group the nitrogen atoms were also found to take part in hydrogen bonding. However, the roles of other interactions are not so easy to distinguish but the surface of the binding pocket of the receptor is mainly hydrophobic. It is assumed that the activity of the agonists is affected not only by a geometric complementarity between the ligand guests and protein pocket hosts, but also by the ability of the ligands to form stable supramolecular associates by means of specific hydrogen bonding and van der Waals interactions with the macromolecules. The results of both X-ray and theoretical studies have shown that hydrogen bonds and van der Waals interactions are, at least, partially responsible for the ligand binding with the protein chain (Andrews *et al.*, 1984 ▸; Carver *et al.*, 1998 ▸; Freitas *et al.*, 2010 ▸).

We believe that the crystal packing of a molecule can provide valuable information about trends in its supramolecular organization, disposition of the active sites, and hydrophobic and hydrophilic regions. Experimental charge density studies of various biologically active species are powerful instruments to gain insight into the mechanism of those pharmacological activities. Information about charge distribution, weak intermolecular interactions and dipole moments derived from high-resolution X-ray studies is relevant to molecular recognition processes, particularly to describe and understand bonding between a compound of interest and the active site of a receptor (Dittrich & Matta, 2014 ▸; Malinska *et al.*, 2015 ▸, 2014 ▸). According to Pinkerton and coworkers, the binding affinity of estrogen (Parrish *et al.*, 2006 ▸; Yearley *et al.*, 2008 ▸; Zhurova *et al.*, 2016 ▸) to the particular sites of the receptor can be predicted using some functions of molecular electrostatic potential (MEP). The distributions of MEP functions derived from high-resolution X-ray studies can be sufficient to evaluate the nature and strength of the ligand–receptor binding. Some useful information about the binding affinity of **Bic** can be obtained using more sophisticated approaches such as quantum theory of atoms in molecules (Bader, 1990 ▸) (QTAIM), non-covalent interaction analysis (NCI) (Johnson *et al.*, 2010 ▸; Saleh *et al.*, 2012 ▸) and reduced density gradient (RDG). Our work is focused on the evaluation of these quantities from high-resolution X-ray experiments and applications of these parameters to study the binding of the receptor to different sites.

## 2. Experimental   

### 2.1. Data collection and reduction   

Single crystals were grown at room temperature from commercially available racemic **Bic** by slow evaporation from ethanol. Single crystals were selected from the precipitate and mounted on a glass needle. The X-ray diffraction dataset was collected at 100 K on an Agilent Super Nova diffractometer equipped with an Oxford Cryostream cooling unit and a microfocus tube with an Mo anode (λ = 0.71073 Å). The omega and phi scans were used at several detector positions, utilizing various exposure times to reach completeness and maintain sufficient redundancy at high diffraction angles. The measured intensities were integrated and corrected for absorption using the *CrysalisPRO* software (Agilent Technologies Ltd, 2014 ▸).

### 2.2. IAM refinement   

The crystal structure was solved using *SHELXT* (Sheldrick, 2015*b* ▸) and refined with *SHELXL* (Sheldrick, 2015*a* ▸) and *OLEX2* (Dolomanov *et al.*, 2009 ▸). All hydrogen positions were calculated and refined using the riding model. The structure of racemic **Bic** was the same as the one described in the work published by Hu & Gu (2005 ▸).

### 2.3. Multipole refinement   

The charge distribution for a single crystal of **Bic** was obtained by applying the multipole formalism (Hansen & Coppens, 1978 ▸) as implemented in the *XD* package (Koritsansky *et al.*, 2003 ▸) with the core and valence electron density derived from wavefunctions fitted to a relativistic Dirac–Fock solution. In the first step, the scale factor was refined on all data. Next, a high-order refinement (sin(θ)/λ > 0.7 Å) of atomic positions and atomic displacement parameters of all non-hydrogen atoms was employed followed by refinement of hydrogen atom positions, with the C—H, N—H and O—H distances fixed at values taken from neutron diffraction (Allen & Bruno, 2010 ▸). Then, the ADPs for the hydrogen atoms were estimated using the *SHADE3* software (Madsen, 2006 ▸). The multipolar expansion was truncated at the hexadecapolar level for S(1) and O(4) atoms, and at the octapolar level for the other non-hydrogen atoms. For the hydrogen atoms, only the monopole and dipole populations in the bond directions were refined. The κ and κ′ values were kept fixed to the theoretical values for the hydrogen atoms (Volkov *et al.*, 2001 ▸). Individual κ and κ′ parameters were refined for the fluorine atoms of the CF_3_ and C_Ph_–F moieties, for the two nitrogen atoms, for the oxygen atoms of different groups and for the several carbon atoms (*e.g.* *ipso*-atoms of the Ph rings). In total, 12 κ parameters were utilized. The anharmonic nuclear motion with third- and fourth-order Gram–Charlier parameters were refined for the S(1) and O(4) atoms (see below) against sin(θ)/λ > 0.7 Å data. This removed the shashlik-like pattern of the residual density isosurface typical for unmodeled anharmonic motion (Meindl *et al.*, 2010 ▸) for the S(1) atom and the C=O group. In all subsequent steps, the non-zero Gram–Charlier coefficients were fixed at the obtained values and the other coefficients were set to zero. At the final stage of refinement, all multipole parameters, positions and thermal parameters of all non-hydrogen atoms and monopoles [except for the S(1) atom] were refined. All bonded pairs of atoms satisfied the Hirshfeld criterion. Parameters of the experiment and refinement are listed in Table 1 ▸. To evaluate the quality of the model, maps of deformation electron density were drawn (Fig. S6) and included in the supporting information. Also, the residual electron density maps, analysis of the residual density according to Meindl & Henn (2008 ▸, 2014 ▸) and the *DRK-plot* (Zhurov *et al.*, 2008 ▸) obtained via the *WinGX* suite (Farrugia, 2012 ▸) are given and discussed in the supporting information.

### 2.4. Lattice energy computations   

Lattice energy calculations were performed using *CRYSTAL17* code (Dovesi *et al.*, 2018 ▸). The structures were optimized with the dispersion corrected B3LYP-D3(BJ) (Grimme, 2011 ▸; Grimme *et al.*, 2010 ▸, 2011 ▸) hybrid functional and the 6–31G** basis set. The results were corrected for the basis set superposition error (BSSE). Ghost atoms used for the BSSE estimation were selected up to 5 Å distance from the considered molecule in the crystal lattice. The unit-cell parameters were fixed with lattice parameters determined from the X-ray diffraction experiments, allowing only the atomic coordinates to vary during the optimization.

### 2.5. Graphical representation   

Molecular graphics were drawn using the program *OLEX2*. The surfaces of the RDG and MEP functions were calculated using *XDPROP* from the *XD2016* package (Volkov *et al.*, 2016 ▸). The RDG surfaces were drawn with *ChemCraft* (Zhurko & Zhurko, 2011 ▸) and the MEP ones were visualized with *PyMOL*.

### 2.6. Electrostatic calculations (ligand–protein complexes)   

Pseudo-atom data banks allow for reconstruction of electron density of macromolecular systems for which experimentally derived geometries are available. In this study, we used the University at Buffalo Databank (UBDB; Jarzembska & Dominiak, 2012 ▸) together with the program *LSDB* to transfer the multipole parameters of the atom types stored in the UBDB for the protein–**Bic** complexes.

### 2.7. Preparation of protein structures   

**Bic** was found in ten PDB entries (Berman *et al.*, 2000 ▸). The criteria used in model selection were: data resolution (better than 2.5 Å); number of missing atoms of main and side chains, model quality indicators, *i.e.* *R*_free_; clashscore; Ramachandran outliers; side-chain outliers and RSRZ outliers. After applying the selection criteria, four crystal structures with **Bic** bound to the ligand binding domain of the AR W741L mutant [W741L-AR-LBD, PDB entries: 4ojb, 4ok1, 4okx, Hsu *et al.* (2014 ▸); 1z95, Bohl *et al.* (2005 ▸)] and one bound to human serum albumin [PDB entry: 4la0, Wang *et al.* (2013 ▸)] were considered for further study. For all the analysed PDB structures, first we used the *Chimera* software (Pettersen *et al.*, 2004 ▸) to add hydrogen atoms to water molecules, protein residues and ligands to optimize the hydrogen bond network, and then the water molecules were removed. For the **Bic**:AR complexes, two water molecules that were parts of the binding pocket were left. Arg, Lys, Asp and Glu residues were treated as ionized, assuming the ligand binds at pH 7. All amino-acid residues and molecules of **Bic** were scaled independently to their formal charges after the data bank transfer. *X*—H hydrogen bond lengths were extended to the standard neutron diffraction values (Allen & Bruno, 2010 ▸) and fixed in the case of all performed calculations.

### 2.8. Electrostatic interaction energy between ligand and protein   

To obtain the electrostatic interaction energy (*E*_el_) between drug and receptor, the exact potential and multipole model (EP/MM) (Volkov *et al.*, 2004 ▸) was applied, which allowed computation of *E*_el_ between two molecular charge distributions represented within the Hansen–Coppens electron-density formalism. It combines a numerical evaluation of the exact Coulomb integral for short-range interatomic interactions (less than 4.5 Å) with a Buckingham-type multipole approximation for the long-range contacts. After generating charge density distributions of selected complexes with the aid of the UBDB, the EP/MM method was executed in *XDPROP* (Volkov *et al.*, 2016 ▸). Human serum albumin consists of two independent protein chains in the asymmetric unit; the chains were analysed separately.

### 2.9. Electrostatic potential analysis   

All MEPs were calculated using *XDPROP* (Volkov *et al.*, 2016 ▸) and visualized in *PyMOL*. The charge-density distribution for all studied kinases was reconstructed with the aid of the UBDB; this reconstruction was also performed for the electrostatic energy calculations (Jarzembska & Dominiak, 2012 ▸). The terminal residues were completed by hydrogen atoms or methyl groups to achieve chemically sensible groups and a formal charge of the residues. The MEP of the AR (PDB entry: 1z95) was calculated without the ligand in the binding pocket.

## 3. Results and discussion   

### 3.1. QTAIM analysis of **Bic** in the crystalline state   

**Figure 2:**
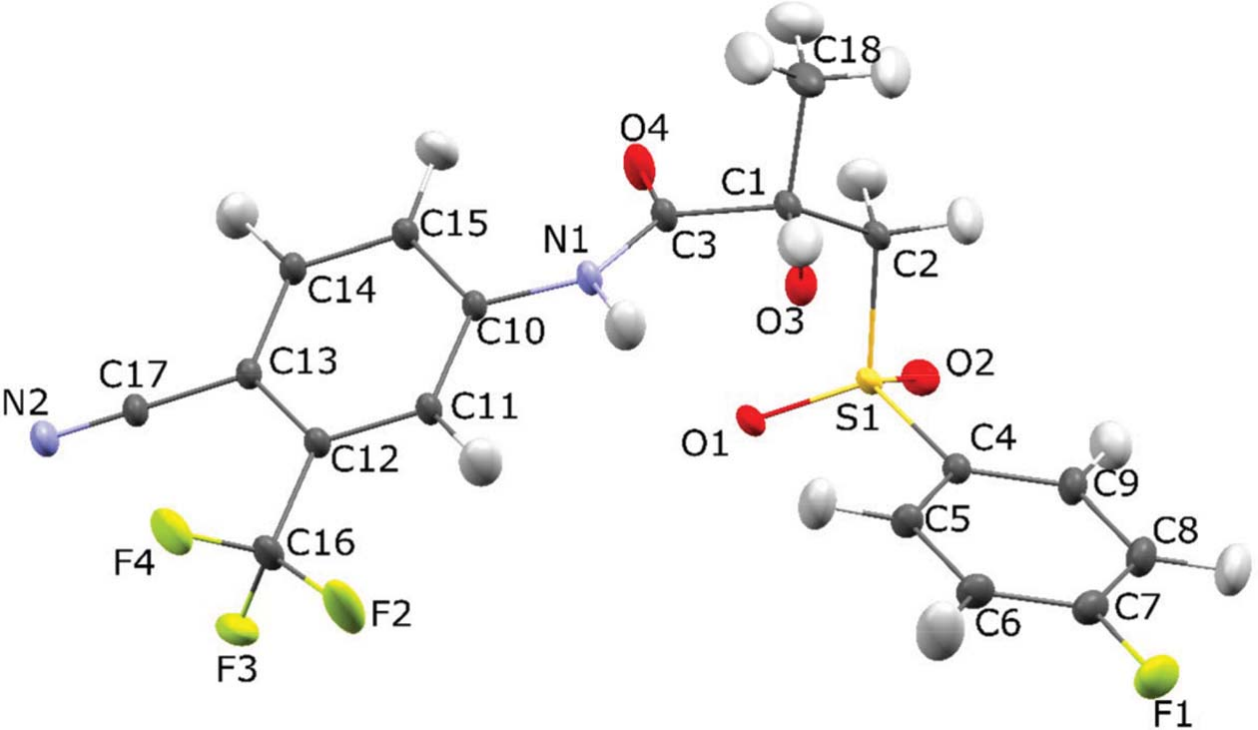
Molecular structure of **Bic**. Atoms are drawn as ADP ellipsoids at *p* = 50% probability level.

**Figure 3:**
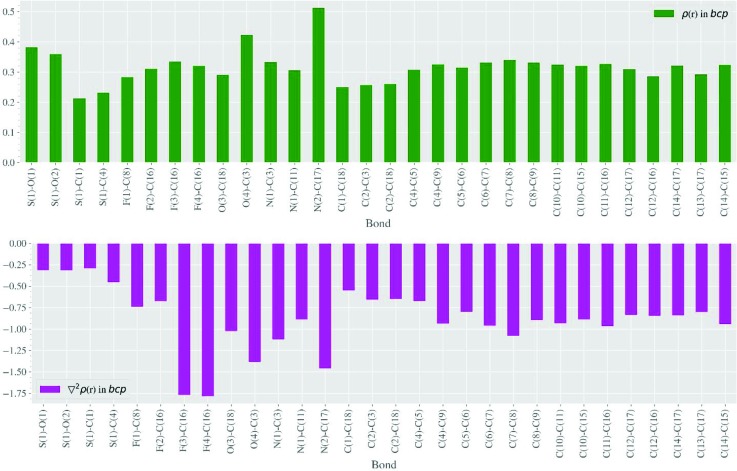
Values of electron density (upper diagram) and its Laplacian (lower diagram) at bcps in the **Bic** crystal structure (a.u.).

The **Bic** molecule (Fig. 2 ▸) contains diverse chemical bonds; therefore, numerous intermolecular contacts of various types were identified. Bond critical points (3, −1) (bcps) and molecular graphs of these can be found in the supporting information. To estimate the energy of the classical and other intermolecular interactions we used empirical correlations as proposed by Espinosa, Mollins & Lecomte (1998 ▸) (EML). As expected, the values of the electron density [ρ(**r**)], its Laplacian [∇^2^ρ(**r**)] and the bond ellipticity at bcps are in agreement with the bond order evaluated from the corresponding bond lengths. The bcp information is summarized as a column diagram in Fig. 3 ▸ and in Table S1 of the supporting information. For instance, the value of ρ(**r**) in the case of the C12—C13 bond is smaller than those for other aromatic C—C bonds, because the C12 and C13 atoms are bonded with two strong acceptor substituents (-CF_3_ and -C≡N). Almost all bcps corresponding to chemical bonds are characterized by a negative-sign Laplacian which is typical for covalent bonds formed by C, N and O atoms in organic compounds. S—O bonds also have a negative sign for ∇^2^ρ(**r**) and the same was observed in several experimental charge density studies of inorganic compounds. On the other hand, in some experimental charge-density studies, as well as in the case of quantum chemical calculations, a positive sign for ∇^2^ρ(**r**) was obtained. Sections of ∇^2^ρ(**r**) and the deformation electron density (Fig. S6) showed that the electron density in the region of the S—O bond was shifted towards the oxygen atom, thus indicating its polar character.

Although in the crystal structure the **Bic** molecule exists in a conformation different from that observed in all other ligand–protein complexes, it is obvious that the OH group participates in a hydrogen bonding interaction with the carbonyl atom of the adjacent molecule, with an O(3)⋯O(4) distance of 3.1235 (6) Å, which is comparable to that of the complex of **Bic** and the receptor CYP46A1. The strength of this bond in the monoclinic polymorph of **Bic** is rather low (−10.5 kJ mol^−1^) based on the EML method (Table 2 ▸); however, it is the strongest intermolecular interaction formed by the **Bic** molecule. The energies of hydrogen bonds N—H⋯O, C—H⋯O, C—H⋯N and C—H⋯F (Table 2 ▸) do not exceed −10.0 kJ mol^−1^ (see Table S3 for further details). Notably, the cyan group, which can be a strong hydrogen bond acceptor in complex, participates only in C—H⋯N interactions with calculated energies up to −6.3 kJ mol^−1^. Note, that the energies of the hydrogen bonds behave as expected by rationalization of the competing hydrogen bond donors and acceptors and are estimated using a hydrogen-bonding propensities tool (Galek *et al.*, 2009 ▸, 2007 ▸). It is expected that the hydroxyl group is more likely to be a hydrogen bond donor than the amide group, whereas the oxygen atom of the amide group is as likely to be an acceptor of a hydrogen bond as the sulfonyl group or the nitrile fragment, but exceeds that of the hydroxyl group. In particular, the propensities of O—H⋯O=C interactions in **Bic** and the O—H⋯N≡C and N—H⋯O=S interactions found in its triclinic polymorph [CSD entry: JAYCES02] are equal to 0.40, 0.39 and 0.29, respectively.

The stacking contacts have dispersive character, and only two bcps were found between the C(7) or C(15) atoms of parallel rings with *E* = −2.6 or −1.8 kJ mol^−1^, respectively. For the 4-PhF ring, this stacking is additionally supported by two F(1)⋯O(3) interactions which may be as strong as *ca* −2.1 kJ mol^−1^. The total energy of intermolecular interactions estimated from the EML correlation is equal to −201.4 kJ mol^−1^. The latter value is very close to the value of −203.4 kJ mol^−1^ obtained for the total packing energy calculated using the ‘UNI’ force-field (Gavezzotti, 1994 ▸; Gavezzotti & Filippini, 1994 ▸). Besides, it is comparable with the binding energy of **Bic** to various regions of AR (*E*_vdw_) which is in the range −243.2 to −217.8 kJ mol^−1^ (Liu *et al.*, 2016 ▸) according to MD simulations. Thus, van der Waals interactions play a primary role in the crystal packing of **Bic**, especially taking into account that in previously reported complexes of **Bic** with receptors, the **Bic** molecule is observed in a conformation with an intramolecular O—H⋯O or N—H⋯O bond in such a way that only one H-donor group is able to take part in hydrogen bonding with the macromolecule.

### 3.2. Analysis of non-covalent interactions in terms of the NCI method   

A more comprehensive description of intermolecular bonding in **Bic** can be provided by the NCI method utilizing the quantity RDG = |∇ρ(**r**)|/2(3π^2^)^1/3^ρ(**r**)^2/3^. The sign of the eigenvector λ_2_ serves as a descriptor of the nature of the non-covalent interactions (attractive or repulsive). The isovalue of RDG and the value of sign(λ_2_)ρ (electron density multiplied by the sign of the λ_2_ eigenvalue) were used in our study to reveal the character of weak intermolecular bonds, especially H⋯H ones. The presence of separate isosurfaces in the regions of small values of ρ(**r**) and its gradient are indicative of weak interatomic bonds and can be described as analogous to bcps. The areas of negative values of sign(λ_2_)ρ are indicative of attractive interactions responsible for the stabilization of a particular atomic configuration or crystal structure. On the contrary, positive values of sign(λ_2_)ρ can be interpreted as the presence of interactions that have repulsive character resulting in destabilization.

**Figure 4:**
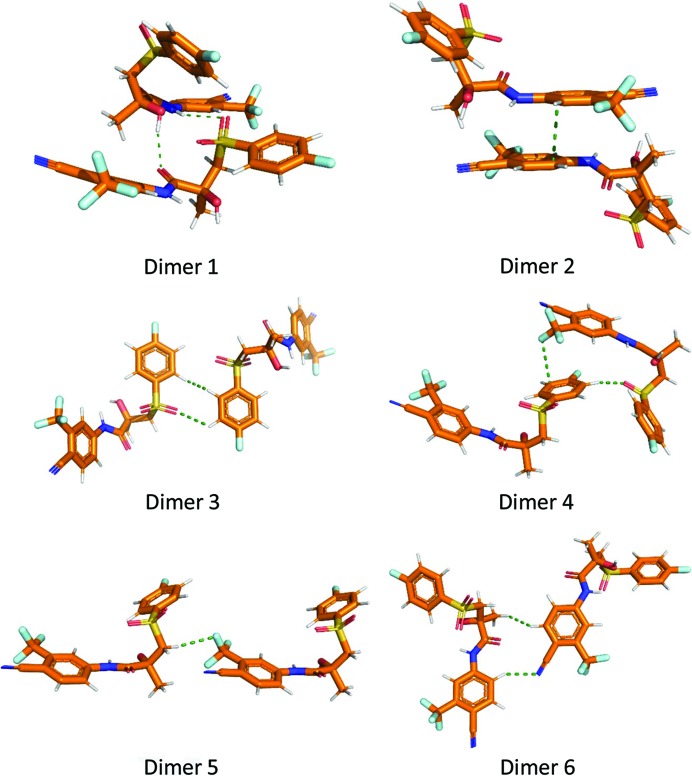
Visualization of the strongest dimer interaction extracted from the crystal structure of **Bic**.

**Figure 5:**
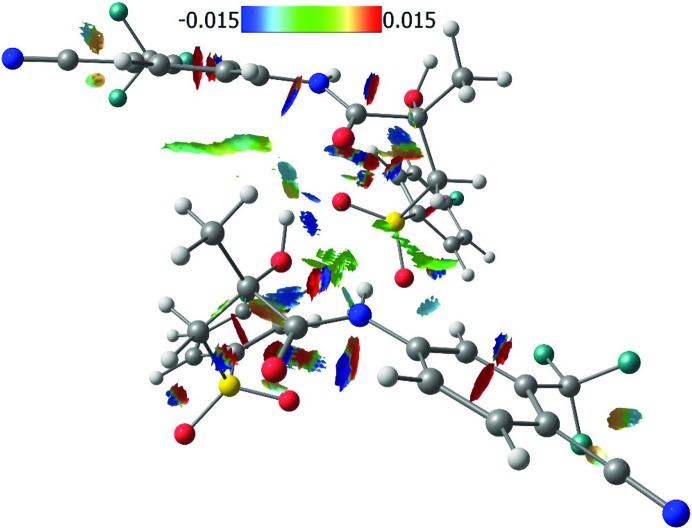
RDG isosurfaces (0.4 a.u.) between adjacent molecules (dimer 1) related to hydrogen bonds in **Bic**. The *x*, −*y* + 1/2, *z* + 1/2 symmetry operation is used to generate the adjacent molecule. The colour scale represents the value of sign(λ_2_)ρ.

In contrast with QTAIM data, the NCI analysis demonstrated the presence and delocalized nature of the π⋯π stacking interactions between substituted phenyl rings in the crystal packing of **Bic**. The areas corresponding to stabilization in this case are comparable with those for destabilization. Separate isosurfaces are also clearly visible for intra- and intermolecular hydrogen bonds and in the case of the non-classic H⋯H and C—H⋯π bonds. The most pronounced difference between classic hydrogen bonds and other types of interatomic interactions is illustrated by the surface area. We studied the three strongest interactions between pairs of molecules: dimer 1, dimer 2 and dimer 3 (Fig. 4 ▸). In dimer 1 the hydrogen bonds and H⋯H interactions play a significant role. In dimer 2 and dimer 3 the parallel orientation of the Ph rings indicates the significant contribution of the stacking interaction for the corresponding interaction energy. The RDG isosurfaces for these dimers are shown in Figs. 5 ▸ and 6 ▸. The values of the electron density in the regions of the RDG isosurfaces correspond to O⋯H bonds (small oblate isosurface in blue, Fig. 5 ▸), which are larger than those for π⋯π stacking, H⋯H or C—H⋯π bonds. At the same time, these interactions have large surface areas, so their role can be underestimated from the point of view of conventional QTAIM analysis.

### 3.3. Pairwise interactions in crystal packing   

QTAIM, MEP and NCI analyses carried out in the conventional way provide no direct information about the energies of intermolecular interactions. The application of the EML correlation to the evaluation of interatomic interaction energies is a very attractive way to analyse their strengths, but has obvious limitations related to the uncertainties of the Kirzhnits approximation (Kirzhnits, 1957 ▸) for kinetic energy density. A more solid basis for the calculations of intermolecular interaction energies can be obtained using quantum chemical calculations or reliable empirical potentials. These methods cannot be used for evaluation of separate intermolecular interactions as they were derived from EML correlation calculations. However, the values of the interactions of **Bic** with the nearest surrounding molecules in the crystal packing (pairwise energies) can be obtained and compared with the data from the literature. We chose the method (Mackenzie *et al.*, 2017 ▸) implemented in *CrystalExplorer17.5* software based on the energy decomposition of the wavefunction obtained from CE-B3LYP/6–31G(d,p) calculations of molecular clusters constructed from target molecules and a neighbouring molecule according to the symmetry operations available for particular space groups. As a result, the total energy is broken down into several terms: electrostatic (*E*_el_^CE^), polarization (*E*_pol_), dispersion (*E*_dis_) and exchange-repulsion (*E*_rep_) energies; these are related to interactions between the charge distributions of individual molecules, the polarization calculated from the charge distribution of molecules, the strength of dispersion forces and the antisymmetric product of the monomer spin orbitals, respectively. All quantities were calculated in terms of the present method, plotted as special diagrams (energy frameworks, see Fig. S7) illustrating the strength and character of the intermolecular interactions between the individual parts. As an alternative, the UNI empirical force field (Gavezzotti & Filippini, 1994 ▸), implemented in *Mercury*, was applied to calculate the intermolecular energies. All calculations were carried out using atomic coordinates from multipolar refinement. The values calculated by the above methods are summarized in Table 3 ▸.

Both methods gave similar results (Table 3 ▸). The energies supplied by the UNI force filed are somewhat higher compared with the CE-B3LYP/6–31G(d,p) calculations. Thus, the values supplied by the latter method were used for further analysis of the intermolecular interactions.

The strength of intermolecular bonding in the **Bic** crystal (−65.2 kJ mol^−1^) was the largest for the interaction with the neighbouring molecule generated by a *x*, −*y* + 1/2, *z* + 1/2 symmetry 2 operation (dimer 1). Indeed, according to QTAIM and NCI analyses, several H⋯O interactions were localized there. The contributions of the electrostatic and dispersion energy terms dominated over those of repulsion and polarization. The energies of interactions of dimer 2 and dimer 4 (−37.9 and −24.1 kJ mol^−1^, respectively) were considerably lower than with the previous cases. In those cases, a parallel orientation of the substituted phenyl rings was observed, so that the contribution of the stacking interaction is noticeable. A QTAIM study revealed the absence of bcps between these rings; however, NCI analysis showed that the corresponding interactions are mostly attractive in nature. It is noteworthy that the dispersion term for the first dimer dominated over the others. The electrostatic and dispersion terms for the third instance of intermolecular bonding were almost equal.

### 3.4. Bic:AR complexes   

The crystal structure of the AR ligand binding domain (LBD) was first solved by Matias *et al.* (2000 ▸), and subsequently, many other structures of the complex were deposited into the PDB. To facilitate the purification and crystallization of the AR significantly, a Trp741Leu complex mutation of the Trp-741 to Leu was introduced to investigate a possible agonist conformation. The three-dimensional structure was arranged in a three-layer, antiparallel α-helical sandwich fold that is characteristic of NR LBDs. The AR LBD consists of eleven α-helices (H) and four short β-strands forming two anti-parallel β-sheets. There is an LBP surrounded by the H3, H5 and H11 atoms of the N termini. The H12 atom, which forms the core of the activation function 2 domain (AF2), acts as a lid to close the LBP upon agonist binding.

**Figure 7:**
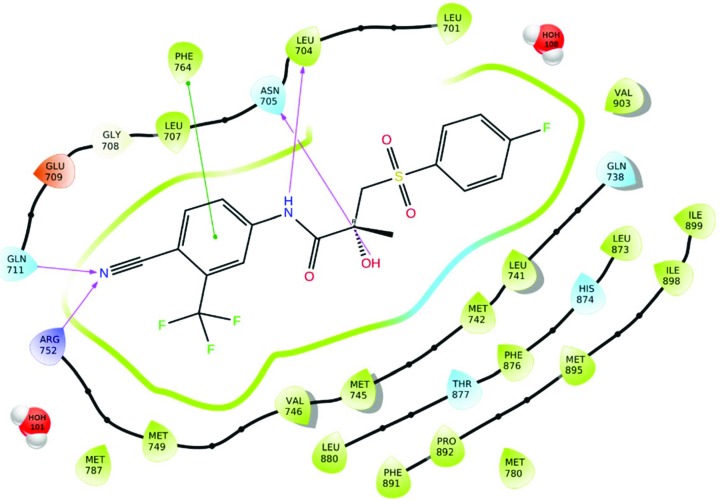
Schematic representation of the interactions between **Bic** and the residues of the AR receptor in LBD (PDB entry: 1z95). The colours of the residues indicate their character: purple – charged (positive), red – charged (negative), white – glycine, blue – polar, and green – hydrophobic. Lines connecting the atoms of the ligand and residues represent these interactions: violet arrow – hydrogen bond, green line – π⋯π stacking.

**Figure 8:**
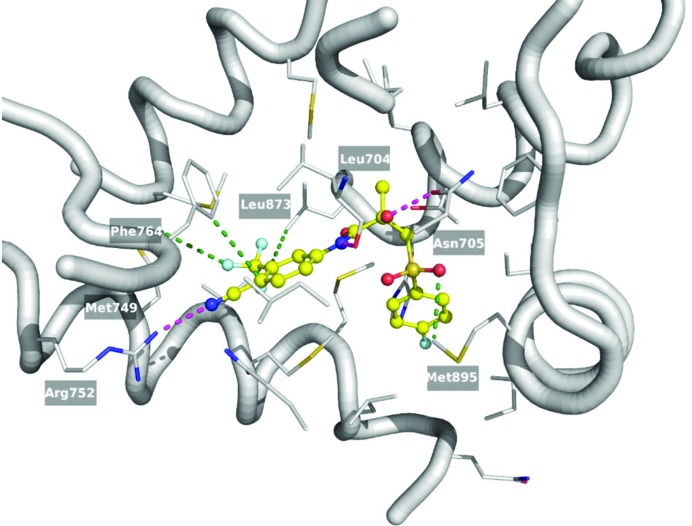
**Bic** (shown as yellow balls and sticks) in the binding pocket of the AR LBD (PDB entry: 1z95). Selected side chains of protein residues are represented as grey sticks; the rest of the protein backbone is shown as a ribbon. The strongest interacting residues are labelled and the closest contacts to the ligand atoms are shown as dashed lines: magenta for a classic hydrogen bond, green for any other important electrostatic interaction.

**Bic** in pharmaceutical products is available as a racemic mixture; however, the *R* isomer has a *ca* 30-fold higher binding affinity to the AR than the *S* isomer (Mukherjee *et al.*, 1996 ▸). Only the *R* isomer was crystalized as a complex with AR. Hydrogen bonds were present between *R*-**Bic** and the AR binding pocket in two different regions (Fig. 7 ▸). The first consisted of the *A* ring cyan group of *R*-**Bic** and was located at a distance of *ca* 3.0 Å from Arg-752 Nη2, which indicated a possible hydrogen bonding interaction. In all four complexes, *E*_el_ with Arg-752 was around −64 kJ mol^−1^ (Table 4 ▸), clearly confirming the electrostatic character of the contact. Conversely, the Gln-711 N∊2 is further from the cyan group than the Arg Nη2 and may be slightly out of hydrogen bonding range, resulting in an *E*_el_ of around −5 kJ mol^−1^. All the AR structures and the progesterone receptor crystal structures (Matias *et al.*, 2000 ▸; Sack *et al.*, 2001 ▸) have some well conserved water molecules (HOH-1101, HOH-101, HOH-1105 and H-1101 for 4ojb, 1z95, 4ok1 and 4okx, respectively) at a distance of 3.0 Å from the cyan group of the ligand. However, these water molecules form hydrogen bonding interactions with the Arg-752 Nη2, Gln-711 N∊2 and Met-745 O atoms. The *E*_el_ of the ligand with this water molecule is close to −2.3 kJ mol^−1^, thus comprising only a small contribution of the total *E*_el_. Another group that can form stabilizing interactions with the LBP residues are the amide nitrogen and the chiral hydroxyl of *R*-**Bic**. The Leu-704 backbone oxygen forms a contact with the ligand amide nitrogen and the chiral hydroxyl group of *R*-**Bic**, whereas Asn-705 Oδ1 was observed to be closer to the chiral hydroxyl group (Fig. 8 ▸). As a consequence, the *E*_el_ with Leu-704 is close to −29.4 kJ mol^−1^, whereas the second interaction can contribute more significantly to achieving the minimum value for the 1z95 crystal structure (−92.8 kJ mol^−1^). Contrary to steroid-bound AR structures, the Oγ of Thr-877 clearly does not form a hydrogen bond with *R*-**Bic**; nonetheless, the interaction is still stabilizing (*E*_el_ = −7.4 kJ mol^−1^).

As expected from the hydrophobic character of the AR binding pocket, van der Waals forces comprise the majority of interactions between the protein and *R*-**Bic**. The trifluoromethyl group in the *meta* position of the *A* ring is situated in a hydrophobic environment surrounded by Met-742, Val-746, Met-787 and Leu-873. Even though classically hydrophobic in nature, the *E*_el_ between it and the last residue is between −13.6 and −17.4 kJ mol^−1^, suggesting the importance of the electrostatic forces. A large component of these forces arises from the interaction between the trifluoromethyl group and a large negative region of the MEP and the side chain of Leu-873 as well as other contacts involving the *A* ring of *R*-**Bic** including Leu-704, Leu-707, Met-745 and Phe-764. Similar to the previous interaction, here the ring of Phe-764 forms a T-shaped π⋯π stacking interaction, resulting in an *E*_el_ value equal to *ca* −8.8 kJ mol^−1^. The carbonyl oxygen of the amide moiety in *R*-**Bic** lacks any hydrogen bonding partners with the closest atom being the Sδ of Met-742, and under EP/MM analysis exhibits a repulsive interaction from the point of view of electrostatics with the average value for the four structures equal to 11.2 kJ mol^−1^. However, these interactions with Leu-873 are stabilizing (−15 kJ mol^−1^). Again this seems to be the result of the opposing character of the MEP between the ligand and the LBP. In addition, Met-895 comes into close contact with the sulfonyl oxygen atom of *R*-**Bic**. Met-895 also participates in the formation of a hydrophobic pocket enclosing the *B* ring of the ligand along with the other H12 residues Ile-898 and Ile-899, and H5 residues Leu-741 and Met-742. The *E*_el_ for Met-895 has a significant contribution to the total *E*_el_ in the range of a weak hydrogen bond which is between −18.1 and −30.2 kJ mol^−1^. The fluorine atom in the *para* position of the *B* ring however is bound in a more hydrophilic environment, located 2.9 Å from the water molecule (HOH-108 in the 1z95 crystal structure, Fig. 7 ▸). The backbone oxygen atoms of Gln-738 (2.5 kJ mol^−1^), the backbone nitrogen atoms of Leu-741 (−0.3 kJ mol^−1^) and Met-742 (11.2 kJ mol^−1^), and the His-874 N∊2 (−5.0 kJ mol^−1^) are all situated at some suitable hydrogen bonding distances from the same water molecule.

The total *E*_el_ values between **Bic** and the selected residues of the LBD are similar for 4ojb, 1z95 and 4okx (−189.3, −251.6 and −231.0 kJ mol^−1^, respectively). The complex with the highest total *E*_el_ is 4ok1 with a value of −123.8 kJ mol^−1^. This is probably caused by the different orientations of some residue side chains, *e.g.* Gln-711, Met-745 and Thr-877, influencing the charge density of the LBD. Nonetheless the position of the ligand is well conserved in all AR structures.

### 3.5. **Bic** conformers and MEP   

The non-covalent intra- and intermolecular bonds found in crystals of **Bic** can provide the initial information about the ability of molecular fragments of **Bic** to bind to protein chains of the receptors and their molecular shapes can give information about their complementarity. However, **Bic** is a very flexible molecule with a conformation defined by the rotation around the S(1)—C(1), C(1)—C(2), C(1)—C(3), C(3)—N(1), N(1)—C(10) and C(2)—O(3) bonds. Depending on the environment, the molecule changes its conformation and, as a result, its charge density, electrostatic potential and molecular shape. Dhaked *et al.* reported a total of 18 **Bic** rotamers that lie within an energy range of 50.2 kJ mol^−1^ in the gas phase (Dhaked *et al.*, 2012 ▸). Those having relative energies below ∼12.6 kJ mol^−1^ are stabilized by two strong intramolecular hydrogen bonds between the hydrogen atom of the hydroxyl group and the oxygen atom of the sulfonyl group, and the second bond, between the hydrogen atom of the amide group and the oxygen atom of the hydroxyl group. The absence of such hydrogen bonding interactions results in a 17–50 kJ mol^−1^ greater relative energy. However, solvent calculations have suggested that a polar solvent strongly stabilizes the conformer lacking the first hydrogen bond.

**Figure 9:**
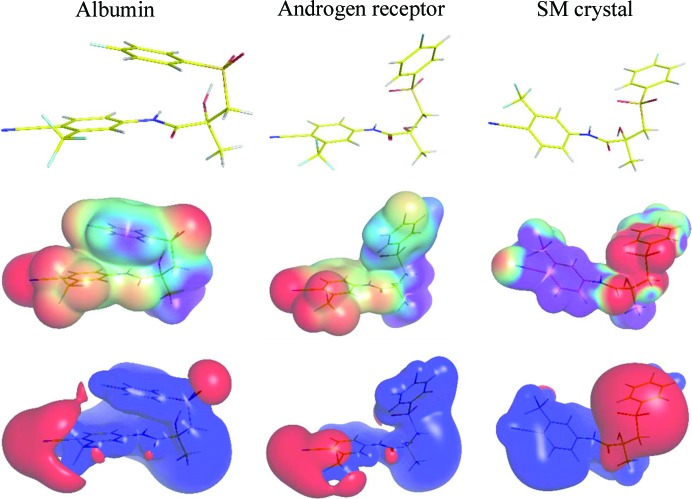
Conformations of **Bic** (top row), molecular electrostatic potential mapped on the 0.068 e Å^−3^ isosurface of the charge density (middle row) and the electrostatic potential isosurfaces of 0.05 e Å^−1^(blue) and −0.05 e Å^−1^ (red) (bottom row) from the **Bic**:albumin complex (PDB entry: 4la0, left column), the **Bic**:AR complex (PDB entry: 1z95, middle column) and the molecular crystal (right column).

As expected, the negative values of MEP are located near the sulfonyl group and fluorine atoms for all conformations (Fig. 1 ▸), thus indicating that these sites are suitable hydrogen bond acceptors (Fig. 9 ▸). The H(1) and H(3) atoms can be H-donors because the values of the MEP around these sites have positive values. The **Bic** molecule forms an intramolecular hydrogen bond and an internal π⋯π stacking interaction which stabilizes the conformation present in the albumin binding pocket that also has hydrophobic character. The only stabilizing interaction present is between the cyan group of **Bic** and Lys-137 (Table S4) which secures the placement of the ligand. The internal charge separation (Π) for this conformation is 0.048 e Å^−1^ (Table 5 ▸), showing both a significant charge separation and the increased polarity of the molecule. A **Bic** molecule in complex with the AR has a different conformation than with the previous one because of the rotation about the C(1)—C(3) single bond. The most negative part of the MEP is located at the same place; however, as the intramolecular interactions are broken, the **Bic** molecule can form contacts with the AR binding pocket. The strongest interactions that are are probably responsible for the ligand placement are the hydrogen bonds to Leu-704, Asn-705 and Arg-752 that sum to an *E*_el_ of around 150 kJ mol^−1^; this constitutes 3/4 of the total energy. Comparing the statistical quantities that characterize the MEP shows that the conformational change has not influenced the MEP characteristics. However, moving from the protein polar environment to a small-molecule crystal structure, Π rises three times to 0.144 e Å^−1^. The main reason being that a conformational change of the PhF ring brings the sulfonyl and amide groups closer together, moving the negative MEP parts towards the same location. Moreover, the PhCN ring is flipped which results in the localization of the positive MEP near the ring. The relative strengths of the positive and negative surface potentials (υ, Table 5 ▸) reached a maximum of 0.224.

In fact, all these observations are in good agreement with the structures of ligand–receptor complexes. As a result of the charge-density distribution in the monoclinic polymorph, **Bic** bears a dipole moment of *ca* 23.7 D. Ligand conformational changes improve the fit into the binding pocket in such a way as to form more stabilizing interactions. The highest contribution to the total interaction energy arises from an amide group through its contacts in the monoclinic polymorph (dimer1, −71.6 kJ mol^−1^) and in the AR, on average −83.6 kJ mol^−1^ (Asn-705, Leu-704). Not only the strongest interactions are conserved, but the trifluoromethyl group interacts with the methylene group of the closest molecule (dimer 5), and also in the protein complex, interacting instead with Met-749 (−24.5 kJ mol^−1^). The biggest difference is found in the interaction of the cyan group which, in the AR binding pocket, forms a strong directional hydrogen bond with Arg-752; however, in the polymorphic structure, the cyan group is surrounded by phenyl rings and plays a secondary role in π⋯π stacking interactions.

**Figure 10:**
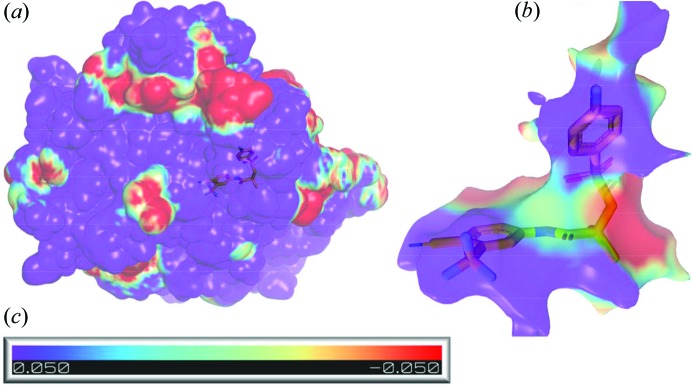
Electrostatic potential (e Å^−1^) mapped onto the density isosurface (0.067 e Å^−3^) for (*a*) AR and (*b*) the binding site of AR. (*c*) ESP scale for all figures. The ESP of AR was calculated without the ligand in the binding pocket. Ligands are shown in stick representation to orient the binding pocket.

The MEP of the AR is in majority positive with only small patches of negative values above the ligand. [Fig. 10 ▸(*a*)]. The binding pocket, considered hydrophobic, has rather polar character with positive MEPs at the extremes and negative ones at the middle [Fig. 10 ▸(*b*)]. The MEP of the binding pocket, calculated without a ligand, showed good complementarity between the two moieties. The maximum value of the MEP of the binding pocket is located at the bottom (Arg-752) where the minimum of the ligand MEP is present. The middle part of the ligand also fits nicely into the binding pocket. The less fitted is the last part (PhF ring) where two positive MEP surfaces are placed. The binding pocket MEP and its overall shape explain why none of the molecule lowest conformations can bind to the AR.

This similarity can also be observed in the total *E*_el_. The electrostatic lattice energy calculated by the EP/MM method based on the experimental charge density was calculated to be −210.5 kJ mol^−1^, which is very close to the averaged electrostatic interaction energy in the AR binding pocket, −198.9 kJ mol^−1^. However, the total lattice energy of the monoclinic polymorph based on periodic DFT calculations is −498.5 kJ mol^−1^, highlighting the importance of dispersive interactions in the crystal structure that play an important role in the binding interactions. The lattice energy for the triclinic polymorphs is −497.8 kJ mol^−1^. Nevertheless, the role of electrostatic interactions in the stabilization of crystal packing and the ligand–receptor complex can be suitably investigated using EP/MM, QTAIM and NCI methods.

## 4. Conclusions   

The non-steroidal drug bicalutamide is on the World Health Organization’s List of Essential Medicines. Crystal structures of two polymorphs, several co-crystals and the **Bic** protein complexes showed vast molecular flexibility, confirmed by quantum chemical calculations and MD simulations. Although the formally single bonds connect two phenyl rings in the molecule, their conformation is rather rigid. The lowest energy conformation of the drug with two intramolecular hydrogen bonds was found in the complex with albumin. In different environments the orientation of the phenyl-CF_3_ ring changes and behaves like a canopy, whereas the other phenyl ring has two possible orientations. Here, we present an experimental study of the electron-density distribution of **Bic** in its monoclinic polymorph and protein-bound conformation which reveal the conserved nature of the molecular electrostatic potential and intermolecular bonding based on their propensities and energies. For instance, while bicalutamide bound to the AR exhibits different spatial arrangements, the MEP distribution is unchanged compared with the lowest energy state. The orientation of the hydrogen bond donors and acceptors differ, which allows the formation of the most favourable interactions. This conformation complements the MEP of the binding pocket in a constructive way. In terms of hydrogen bonding propensity, the most likely interaction, the hydroxyl–amide pair, was also found to be the strongest of all the intermolecular interactions found in the monoclinic structure and in the AR complexes. The conformational angle of the nitrile group of the drug molecule causes the formation of numerous C—H⋯N≡C interactions in the crystal structure, and its interaction with Arg-752 is part of the strongest set of interactions in AR complexes. Although numerous, the role of water molecules in the direct stabilization of the drug molecule in the binding pocket was found to be negligible. While these interactions can be classically described as hydrophobic, interactions with Met-749 and Met-895 have significant electrostatic energy values that are probably additionally stabilized by dispersive forces.

Hydrogen bonds and stacking interactions were found to play a crucial role in the formation of the polymorphs and protein complexes. However, we showed that the description of charge density in terms of QTAIM cannot provide all the information that is necessary to describe intermolecular bonding due to their non-directional character. Therefore, this study based on the NCI approach plays a crucial role in understanding the different binding modes of **Bic**.

## Supplementary Material

Crystal structure: contains datablock(s) I. DOI: 10.1107/S2052252519014416/lz5029sup1.cif

Structure factors: contains datablock(s) EXP640. DOI: 10.1107/S2052252519014416/lz5029sup2.hkl

Supporting information. DOI: 10.1107/S2052252519014416/lz5029sup3.pdf

CCDC reference: 1954089

## Figures and Tables

**Figure 6:**
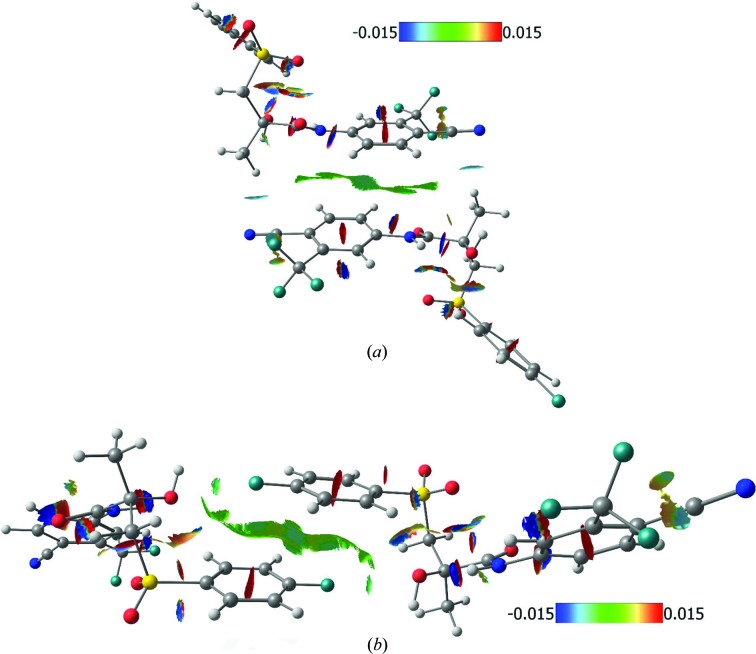
RDG isosurfaces (0.4 a.u) between adjacent molecules (dimer 2 and dimer 3) related to π⋯π stacking interactions between substituted phenyl rings in the **Bic** crystal structure. Symmetry operations used are (*a*) −*x*, 1 − *y*, 1 − *z*. and (*b*) 1 − *x*, 1/2 + *y*, 3/2 − *z*. The colour scale represents the value of sign(λ_2_)ρ.

**Table 1:** Experimental details for the crystal structure of **Bic**

Crystal symbol	**Bic**
Chemical formula	C_18_H_14_F_4_N_2_O_4_S
Formula weight	430.37
Space group	*P*2_1_/*c*
*a*, *b*, *c* (Å)	14.89450 (1), 12.11880 (1), 10.28460 (1)
β (°)	105.8250 (1)
*V* (Å^3^)	1786.05 (3)
*Z*	4
μ (mm^−1^)	0.250
Crystal size (mm)	0.39 × 0.31 × 0.25
*T*_min_, *T*_max_	0.500, 1.000
No. of measured, independent and observed [*I* > 2σ(*I*)] reflections	175282, 9381, 8699
*R*_int_	0.073
(sinθ/λ)_max_ (Å^−1^)	1.111
	
Refinement method IAM/multipole model	
No. of parameters	268/736
Goodness-of-fit	
Spherical atom model (on *F* ^2^)	1.03
Multipole atom model (on *F*)	1.066
Final *R*(*F*) indices [all data]	
Spherical atom model (on *F* ^2^)	*R*_1_ = 0.045, *wR_2_* = 0.110
Multipole atom model (on *F*/*F* ^2^)	*R*_1_ = 0.033/0.02
Final *R*(*F*) indices [*I* > 2σ(*I*)]	
Spherical atom model (on *F* ^2^)	*R*_1_ = 0.038, *wR_2_* = 0.105
Multipole atom model (on *F*/*F* ^2^)	*R*_1_ = 0.02/0.03, *wR_2_* = 0.021/0.041
Δρ_max_, Δρ_min_ (eÅ^−3^)	
Spherical atom model (all data)	1.55, −1.10
Multipole atom model (all data)	0.21, −0.24

**Table 2:** The strongest intermolecular interactions (less than −4.5 kJ mol^−1^) in the crystal structure of **Bic** *V*(**r**) is the potential energy density at the bcp.

Atom 1	Atom 2	*R* (Å)	ρ(**r**)	∇^2^ρ(**r**)	*V*(**r**) (Ha a.u.^−1^)	*E*_bond_ (kJ mol^−1^)
O1	H8	2.255	0.010	0.034	−0.008	−10.0
O2	H11	2.402	0.006	0.030	−0.004	−4.7
O4	H3	2.223	0.012	0.049	−0.008	−10.5
F2	H6	2.432	0.006	0.032	−0.004	−5.2
N2	H15	2.418	0.008	0.036	−0.005	−6.3
N2	H18a	2.665	0.008	0.027	−0.004	−5.4
N2	H1a	2.981	0.009	0.031	−0.005	−6.0

**Table 3:** Energies (kJ mol^−1^) of intermolecular interactions in the **Bic** crystal structure calculated by the UNI force field and the dimer interaction energies calculated using *Crystal Explorer* based on the PIXEL method [CE-B3LYP/6-31 G(d,p)]

		UNI empirical force field	CE-B3LYP/6-31G(d,p)				
Symmetry operation	Dimer	*E*_tot_	*E*_el_	*E*_pol_	*E*_dis_	*E*_rep_	*E*_tot_
*x*, 1/2 − *y*, *z* − 1/2	Dimer 1	−65.2	−42.9	−16.4	−54.0	52.7	−71.6
−*x*, 1 − *y*, 1 − *z*	Dimer 2	−37.0	−16.2	−4.5	−36.6	23.2	−37.9
1 − *x*,1/2 + *y*, 3/2 − *z*	Dimer 3	−29.6	−17.8	−4.3	−13.1	6.8	−29.2
1 − *x*, −*y*, 1 − *z*	Dimer 4	−24.1	−8.8	−4.5	−34.6	24.3	−27.7
*x*, 3/2 − *y*, *z* − 1/2	Dimer 5	−17.4	−12.1	−4.5	−19.9	14.4	−24.5
*x*, − 1 + *y*, *z*	Dimer 6	−16.8	−14.3	−4.6	−15.3	15.1	−22.6

**Table 4:** Electrostatic interaction energies (kJ mol^−1^) between **Bic** and the residues of LBP of the AR calculated by the EP/MM method ARs selected from the PDB for the analysis are 4ojb (2.0 Å), 1z95 (1.85 Å), 4ok1 (2.09 Å) and 4okx (2.1 Å). The numbering of the residues is the same in all four structures, except for water molecules: HOH-1 represents water molecules in proximity to the cyan group, HOH-2 represents a water molecule close to the fluorine atom in the *B* ring. The strongest interactions are highlighted in bold.

		W741L–AR–LBD				
		4ojb	1z95	4ok1	4okx	Average
LEU	701	−0.4	−1.8	25.1	−4.8	4.5
LEU	704	**−35.7**	**−27.6**	**−26.9**	**−27.3**	**−29.4**
ASN	705	**−29.8**	**−92.8**	**−19.5**	**−73.0**	**−53.8**
GLU	706	−7.4	−2.2	−3.5	−2.2	−3.8
LEU	707	1.8	2.8	19.5	4.9	7.2
GLY	708	−3.6	−7.1	−3.8	−8.0	−5.6
GLU	709	−3.4	1.9	1.3	1.2	0.3
GLN	711	−4.0	−4.2	−6.8	−3.9	−4.7
GLN	738	2.6	3.1	2.2	1.9	2.5
LEU	741	0.9	1.5	−4.0	0.2	−0.3
MET	742	13.3	13.6	8.0	10.1	11.2
MET	745	2.5	9.8	6.3	0.3	4.7
VAL	746	−1.9	−2.0	−2.9	−2.5	−2.3
MET	749	**−30.4**	**−30.3**	**−32.7**	**−29.5**	**−30.7**
ARG	752	**−63.7**	**−67.0**	**−58.3**	**−65.5**	**−63.6**
PHE	764	−8.3	−6.0	−11.6	−9.4	−8.8
MET	780	0.3	−1.5	−1.0	−0.3	−0.6
MET	787	6.5	8.9	5.9	7.6	7.2
LEU	873	**−13.9**	**−15.1**	**−17.4**	**−13.6**	**−15.0**
HIS	874	−6.2	−2.9	−8.1	−2.8	−5.0
PHE	876	−2.2	−2.2	−0.9	−2.4	−1.9
THR	877	**−11.6**	**−11.1**	**5.0**	**−11.8**	**−7.4**
LEU	880	4.0	5.4	23.3	5.1	9.5
PHE	891	3.7	4.0	2.3	3.5	3.3
PRO	892	−1.2	−1.9	−1.3	−1.5	−1.5
MET	895	**−18.1**	**−30.2**	**−24.4**	**−19.9**	**−23.2**
ILE	898	−1.9	−2.7	−0.4	−2.0	−1.8
ILE	899	6.7	5.6	−3.9	6.0	3.6
VAL	903	3.8	3.6	2.7	2.9	3.2
HOH1	1101	−1.8	−3.7	−5.5	1.8	−2.3
HOH2	1107	10.1	0.5	7.8	3.9	5.6
		−189.3	−251.6	−123.8	−231.0	−198.9

**Table 5:** Global statistical quantities that characterize the MEP (Politzer *et al.*, 2001 ▸) Vs^+^_av_ – average of the positive surface values, Vs^−^_av_ – average of the negative surface values, Π – average deviation from the average surface value, σ^2^_tot_ – total variance and positive (σ^2^_+_) and negative components (σ^2^_-_). ν defines the relative strengths of the positive and negative surface potentials. Calculation performed with the *MoleCoolQt* program (Hübschle & Dittrich, 2011 ▸).

	SM crystal	Androgen receptor	Albumin
	**Bic**	1z95	4la0
Vs^+^_av_	0.183	0.06	0.063
Vs^−^_av_	−0.119	−0.035	−0.036
Π	0.144	0.048	0.048
σ^2^_+_	0.016	0.0014	0.0013
σ^2^_−_	0.008	0.0004	0.0004
σ^2^_tot_	0.0247	0.0018	0.0017
ν	0.224	0.178	0.177

## References

[bb1] Agilent (2014). *CrysAlisPRO*. Agilent Technologies Ltd, Yarnton, Oxfordshire, England.

[bb2] Allen, F. H. & Bruno, I. J. (2010). *Acta Cryst.* B**66**, 380–386. 10.1107/S0108768110012048 20484809

[bb3] Andrews, P. R., Craik, D. J. & Martin, J. L. (1984). *J. Med. Chem.* **27**, 1648–1657. 10.1021/jm00378a021 6094812

[bb4] Bader, R. W. F. (1990). *Atoms in Molecules: A Quantum Theory*, New York: Oxford University Press.

[bb5] Berman, H. M., Westbrook, J., Feng, Z., Gilliland, G., Bhat, T. N., Weissig, H., Shindyalov, I. N. & Bourne, P. E. (2000). *Nucleic Acids Res.* **28**, 235–242. 10.1093/nar/28.1.235 PMC102472 10592235

[bb6] Bis, J. A., Vishweshwar, P., Weyna, D. & Zaworotko, M. J. (2007). *Mol. Pharm.* **4**, 401–416. 10.1021/mp070012s 17500564

[bb7] Bohl, C. E., Gao, W., Miller, D. D., Bell, C. E. & Dalton, J. T. (2005). *Proc. Natl Acad. Sci.* **102**, 6201–6206. 10.1073/pnas.0500381102 PMC1087923 15833816

[bb8] Bonomo, S., Hansen, C. H., Petrunak, E. M., Scott, E. E., Styrishave, B., Jørgensen, F. S. & Olsen, L. (2016). *Sci. Rep.* **6**, 29468. 10.1038/srep29468 PMC4942611 27406023

[bb9] Carver, F. J., Hunter, C. A., Carver, F. J. & Seward, E. M. (1998). *Chem. Commun.* 775–776.

[bb10] Dhaked, D. K., Jain, V., Kasetti, Y. & Bharatam, P. V. (2012). *Struct. Chem.* **23**, 1857–1866.

[bb12] Dittrich, B. & Matta, C. F. (2014). *IUCrJ*, **1**, 457–469. 10.1107/S2052252514018867 PMC4224464 25485126

[bb13] Dolomanov, O. V., Bourhis, L. J., Gildea, R. J., Howard, J. A. K. & Puschmann, H. (2009). *J. Appl. Cryst.* **42**, 339–341.

[bb15] Dovesi, R., Erba, A., Orlando, R., Zicovich-Wilson, C. M., Civalleri, B., Maschio, L., Rérat, M., Casassa, S., Baima, J., Salustro, S. & Kirtman, B. (2018). *WIREs Comput. Mol. Sci.* **8**, e1360.

[bb16] Espinosa, E., Molins, E. & Lecomte, C. (1998). *Chem. Phys. Lett.* **285**, 170–173.

[bb17] Farrugia, L. J. (2012). *J. Appl. Cryst.* **45**, 849–854.

[bb18] Freitas, F., Sarmento, V., Santilli, C. & Pulcinelli, S. (2010). *Colloids Surf. A Physicochem. Eng. Asp.* **353**, 77–82.

[bb19] Galek, P. T. A., Allen, F. H., Fábián, L. & Feeder, N. (2009). *CrystEngComm*, **11**, 2634–2639.

[bb20] Galek, P. T. A., Fábián, L., Motherwell, W. D. S., Allen, F. H. & Feeder, N. (2007). *Acta Cryst.* B**63**, 768–782. 10.1107/S0108768107030996 17873446

[bb21] Gavezzotti, A. (1994). *Acc. Chem. Res.* **27**, 309–314.

[bb22] Gavezzotti, A. & Filippini, G. (1994). *J. Phys. Chem.* **98**, 4831–4837.

[bb23] Grimme, S. (2011). *WIREs Comput. Mol. Sci.* **1**, 211–228.

[bb24] Grimme, S., Antony, J., Ehrlich, S. & Krieg, H. (2010). *J. Chem. Phys.* **132**, 154104. 10.1063/1.3382344 20423165

[bb25] Grimme, S., Ehrlich, S. & Goerigk, L. (2011). *J. Comput. Chem.* **32**, 1456–1465. 10.1002/jcc.21759 21370243

[bb26] Hansen, N. K. & Coppens, P. (1978). *Acta Cryst.* A**34**, 909–921.

[bb27] Henn, J. & Meindl, K. (2014). *Acta Cryst.* A**70**, 499–513. 10.1107/S2053273314012984 25176997

[bb28] Hsu, C.-L., Liu, J.-S., Wu, P.-L., Guan, H.-H., Chen, Y.-L., Lin, A.-C., Ting, H.-J., Pang, S.-T., Yeh, S.-D., Ma, W.-L., Chen, C.-J., Wu, W.-G. & Chang, C. (2014). *Mol. Oncol.* **8**, 1575–1587. 10.1016/j.molonc.2014.06.009 PMC4253602 25091737

[bb29] Hu, X.-R. & Gu, J.-M. (2005). *Acta Cryst.* E**61**, o3897–o3898.

[bb30] Hübschle, C. B. & Dittrich, B. (2011). *J. Appl. Cryst.* **44**, 238–240. 10.1107/S0021889810042482 PMC3253731 22477783

[bb31] Jarzembska, K. N. & Dominiak, P. M. (2012). *Acta Cryst.* A**68**, 139–147. 10.1107/S0108767311042176 22186290

[bb32] Johnson, E. R., Keinan, S., Mori-Sánchez, P., Contreras-García, J., Cohen, A. J. & Yang, W. (2010). *J. Am. Chem. Soc.* **132**, 6498–6506. 10.1021/ja100936w PMC2864795 20394428

[bb33] Kirzhnits, D. A. (1957). *Sov. Phys. JETP.* pp. 64–72.

[bb34] Koritsansky, T. S., Howard, S. T., Richter, T., Macchi, P., Volkov, A., Gatti, C., Mallinson, P. R., Farrugia, L. J., Su, Z. & Hansen, N. K. (2003). *XD*, *A computer program package for multipole refinement and topological analysis of charge densities from diffraction data.* Free University of Berlin, Germany.

[bb35] Le, Y., Ji, H., Chen, J.-F., Shen, Z., Yun, J. & Pu, M. (2009). *Int. J. Pharm.* **370**, 175–180. 10.1016/j.ijpharm.2008.11.025 19101616

[bb36] Liu, H., Han, R., Li, J., Liu, H. & Zheng, L. (2016). *J. Comput. Aided Mol. Des.* **30**, 1189–1200. 10.1007/s10822-016-9992-2 27848066

[bb37] Mackenzie, C. F., Spackman, P. R., Jayatilaka, D. & Spackman, M. A. (2017). *IUCrJ*, **4**, 575–587. 10.1107/S205225251700848X PMC5600021 28932404

[bb38] Madsen, A. Ø. (2006). *J. Appl. Cryst.* **39**, 757–758.

[bb39] Malinska, M., Jarzembska, K. N., Goral, A. M., Kutner, A., Wozniak, K. & Dominiak, P. M. (2014). *Acta Cryst.* D**70**, 1257–1270. 10.1107/S1399004714002351 24816095

[bb40] Malinska, M., Kutner, A. & Woźniak, K. (2015). *Steroids*, **104**, 220–229. 10.1016/j.steroids.2015.10.007 26476188

[bb41] Mast, N., Zheng, W., Stout, C. D. & Pikuleva, I. A. (2013). *Mol. Pharmacol.* **84**, 86–94. 10.1124/mol.113.085902 PMC3684827 23604141

[bb42] Matias, P. M., Donner, P., Coelho, R., Thomaz, M., Peixoto, C., Macedo, S., Otto, N., Joschko, S., Scholz, P., Wegg, A., Bäsler, S., Schäfer, M., Egner, U. & Carrondo, M. A. (2000). *J. Biol. Chem.* **275**, 26164–26171. 10.1074/jbc.M004571200 10840043

[bb43] Meindl, K. & Henn, J. (2008). *Acta Cryst.* A**64**, 404–418. 10.1107/S0108767308006879 18421130

[bb44] Meindl, K., Herbst-Irmer, R. & Henn, J. (2010). *Acta Cryst.* A**66**, 362–371. 10.1107/S0108767310006343 20404442

[bb45] Mukherjee, A., Kirkovsky, L., Yao, X. T., Yates, R. C., Miller, D. D. & Dalton, J. T. (1996). *Xenobiotica* **26**, 117–122. 10.3109/00498259609046693 8867996

[bb46] Osguthorpe, D. J. & Hagler, A. T. (2011). *Biochemistry*, **50**, 4105–4113. 10.1021/bi102059z PMC3099172 21466228

[bb47] Parrish, D., Zhurova, E. A., Kirschbaum, K. & Pinkerton, A. A. (2006). *J. Phys. Chem. B*, **110**, 26442–26447. 10.1021/jp065638x 17181304

[bb48] Pettersen, E. F., Goddard, T. D., Huang, C. C., Couch, G. S., Greenblatt, D. M., Meng, E. C. & Ferrin, T. E. (2004). *J. Comput. Chem.* **25**, 1605–1612. 10.1002/jcc.20084 15264254

[bb49] Politzer, P., Murray, J. S. & Peralta-Inga, Z. (2001). *Int. J. Quantum Chem.* **85**, 676–684.

[bb50] Sack, J. S., Kish, K. F., Wang, C., Attar, R. M., Kiefer, S. E., An, Y., Wu, G. Y., Scheffler, J. E., Salvati, M. E., Krystek, S. R., Weinmann, R. & Einspahr, H. M. (2001). *Proc. Natl Acad. Sci.* **98**, 4904–4909. 10.1073/pnas.081565498 PMC33136 11320241

[bb51] Saleh, G., Gatti, C. & Lo Presti, L. (2012). *Comput. Theor. Chem.* **998**, 148–163.

[bb52] Sheldrick, G. M. (2015*a*). *Acta Cryst.* C**71**, 3–8.

[bb53] Sheldrick, G. M. (2015*b*). *Acta Cryst.* A**71**, 3–8.

[bb54] Surov, A. O., Solanko, K. A., Bond, A. D., Bauer-Brandl, A. & Perlovich, G. L. (2016). *CrystEngComm*, **18**, 4818–4829.

[bb55] Tan, J., Abrol, R., Trzaskowski, B. & Goddard, W. A. (2012). *J. Chem. Inf. Model.* **52**, 1875–1885. 10.1021/ci300133a 22656649

[bb58] Vega, D. R., Polla, G., Martinez, A., Mendioroz, E. & Reinoso, M. (2007). *Int. J. Pharm.* **328**, 112–118. 10.1016/j.ijpharm.2006.08.001 16978811

[bb59] Volkov, A., Abramov, Y. A. & Coppens, P. (2001). *Acta Cryst.* A**57**, 272–282. 10.1107/s0108767300018547 11326112

[bb60] Volkov, A., Koritsanszky, T. & Coppens, P. (2004). *Chem. Phys. Lett.* **391**, 170–175.

[bb61] Volkov, A., Macchi, P., Farrugia, L. J., Gatti, C., Mallinson, P., Richter, T. & Koritsanszky, T. (2016). *XD2016*. University at Buffalo, State University of New York, NY, USA; University of Milan, Italy; University of Glasgow, UK; CNRISTM, Milan, Italy; Middle Tennessee State University, TN, USA; and Freie Universität, Berlin, Germany.

[bb72] Wang, Z. M., Ho, J. X., Ruble, J. R., Rose, J., Ruker, F., Ellenburg, M., Murphy, R., Click, J., Soistman, E., Wilkerson, L. & Carter, D.C. (2013) *Biochim. Biophys. Acta*, **1830**, 5356–5374. 10.1016/j.bbagen.2013.06.032 23838380

[bb62] Yearley, E. J., Zhurova, E. A., Zhurov, V. V. & Alan Pinkerton, A. (2008). *J. Mol. Struct.* **890**, 240–248.

[bb63] Zhurko, G. A. & Zhurko, D. A. (2011). *CHEMCRAFT*. Version 1.7. http://www.chemcraftprog.com.

[bb64] Zhurova, E. A., Zhurov, V. V., Kumaradhas, P., Cenedese, S. & Pinkerton, A. A. (2016). *J. Phys. Chem. B*, **120**, 8882–8891. 10.1021/acs.jpcb.6b05961 27504698

[bb65] Zhurov, V. V., Zhurova, E. A. & Pinkerton, A. A. (2008). *J. Appl. Cryst.* **41**, 340–349.

