# Supplementary material for: Charge density view on bicalutamide molecular interactions in the monoclinic polymorph and androgen receptor binding pocket
Source: IUCrJ. 2020 Jan 1;7(Pt 1):71–82. doi: 10.1107/S2052252519014416 (PMC6949590; doi:10.1107/S2052252519014416)
Supplement: Supplementary file 3 [file m-07-00071-sup3.pdf]

IUCrJ

Volume 7 (2020)

Supporting information for article:

Charge density view on bicalutamide molecular interactions in the monoclinic polymorph and androgen receptor binding pocket

Alexander A. Korlyukov, Maura Malinska, Anna V. Vologzhanina, Mikhail S. Goizman, Damian Trzybinski and Krzysztof Wozniak

Supporting Information

Charge density view on bicalutamide molecular interactions in monoclinic polymorph and androgen receptor binding pocket

Alexander A. Korlyukov[1‡*], Maura Malinska[2‡], Anna V. Vologzhanina[1], Mikhail S. Goizman[3], Damian Trzybinski[2] and Krzysztof Wozniak[2*]

[1]A.N.Nesmeyanov Institute of Organoelement Compounds of Russian Academy of Sciences, 119991, Vavilov St. 28, Moscow, Russian Federation
[3]Biological and Chemical Research Centre, Department of Chemistry, University of Warsaw, Żwirki i Wigury 101, 02089 Warszawa, Poland
[2]Drug Technology Co, 2a Rabochaya St, Chimki 141400, Moscow Oblast, Russia

Corresponding addresses: akorlyukov@gmail.com and kwozniak@chem.uw.edu.pl

Contents

**Multipole refinement of Bic**	**2**
**Residual electron density distribution**	**3**
**Fractal plot**	**4**
**Normal probability plot**	**5**
**Critical points**	**6**
**Experimental deformation density**	**7**
**Energy framework for crystal packing**	**8**
**Characteristics of intramolecular CPs**	**9**
**The QTAIM integrated parameters**	**11**
**Parameters of intermolecular contacts**	**12**
**Bicalutamide in the binding pocket**	**13**
**Electrostatic interaction energy**	**14**
**References**	**15**

Multipole refinement of Bic.

The model neglecting anharmonic motion for the S(1) atom and the O(4) atom of the carbonyl group (Fig. S1, left) shows the shashlik-like pattern of residual density which is typical for unmodeled anharmonic motion [S1]. It disappears after inclusion of anharmonic nuclear motion in the form of the Gram-Charlier tensors of the third order for the oxygen atom and of the fourth order for the S(1) atom (Fig. S1, right). In order to avoid an abundant parameters refinement only the non-zero Gram-Charlier parameters were refined, while the values of the Gram-Charlier parameters equal to zero within three e.s.d. were set to zero for further refinement. Non-zero values were obtained for seven parameters for the O(4) atom (C^{111}, C^{333}, C^{112}, C^{113}, C^{133}, C^{233}, C^{123}) and three parameters for the S(1) atom (C^{222}, C^{333}, D^{2222}). These Gram-Charlier parameters were refined with the positional and thermal parameters keeping all multipolar parameters fixed. In the next step, only the multipolar parameters were refined. In all further steps of the refinement the above mentioned Gram-Charlier coefficients were fixed at the obtained values and the other coefficients were set to zero.

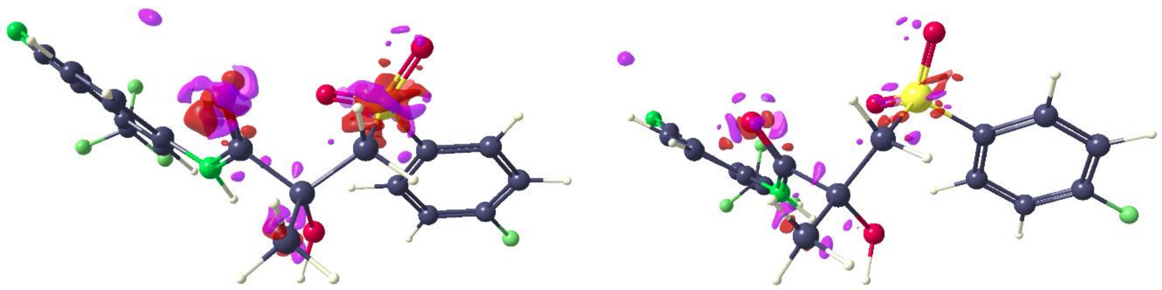

Figure S1 Arrangement of molecules in crystal structure of **Bic** and the isosurface representation (contour value = 0.20 e $Å^{-3}$; red is positive, fuchsia is negative) of the residual density in various refinement models of **Bic**: (left) a model neglecting anharmonic nuclear motion; (right) a model including anharmonic nuclear motion to third order for the O(4) atoms and to fourth order for the S(1) atom.

Residual density distribution.

The residual density maps (Figure S2) for Bic demonstrate that there still remains some small unfitted electron density at S(1) and O(4) atoms that can be accounted for unresolved anharmonic motion. Inspection of the residual density with jnk2RDA developed by Meindl & Henn [S2] results in the distribution shown in Figures S4 and S5. The deviation from the ideal Gaussian shape for 1 originates mainly from the not ideal description of above mentioned atoms. $d^f(0) = 2.71$ is similar with values given in paper describing this procedure suggesting a successful refinement. Broadening of the distribution results in relatively high e_{gross} value and may be accounted for insufficiently accurate s.u. values of intensities measured with CCD area detector. Normal probability plot plotted against full dataset and time scale factor plot vs. the resolution confirms good data quality over the whole resolution range and its internal consistency (Fig. S6) of 1.

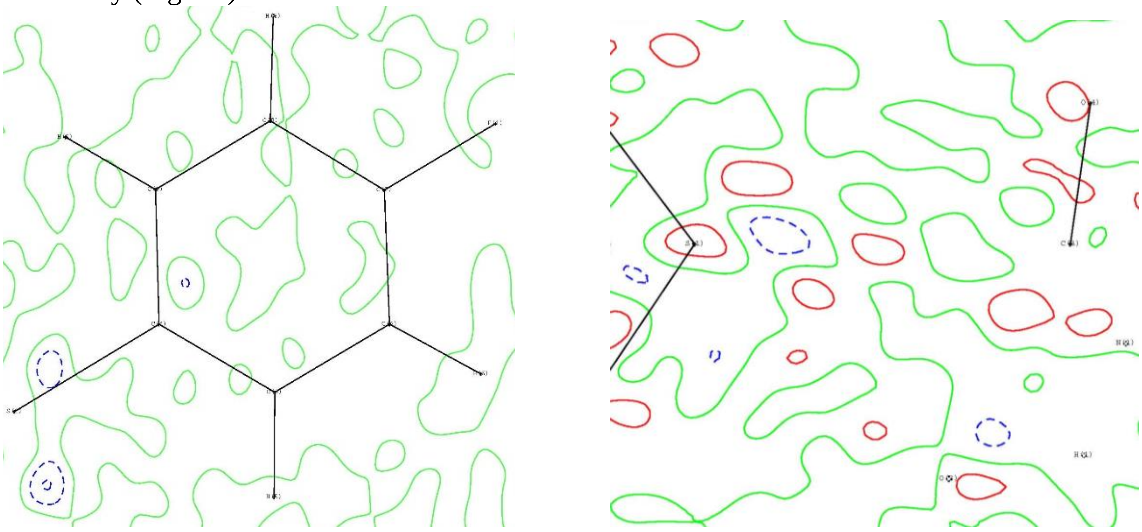

Figure S2 The residual density maps in the sections of (left) C(4), C(6) and C(8); (right) S(1), C(3) and O(4) atoms calculated using all data for **Bic**. Isocontours are drawn every 0.1 e Å^{-3}; positive contours are shown in red, the negative contours are dashed blue.

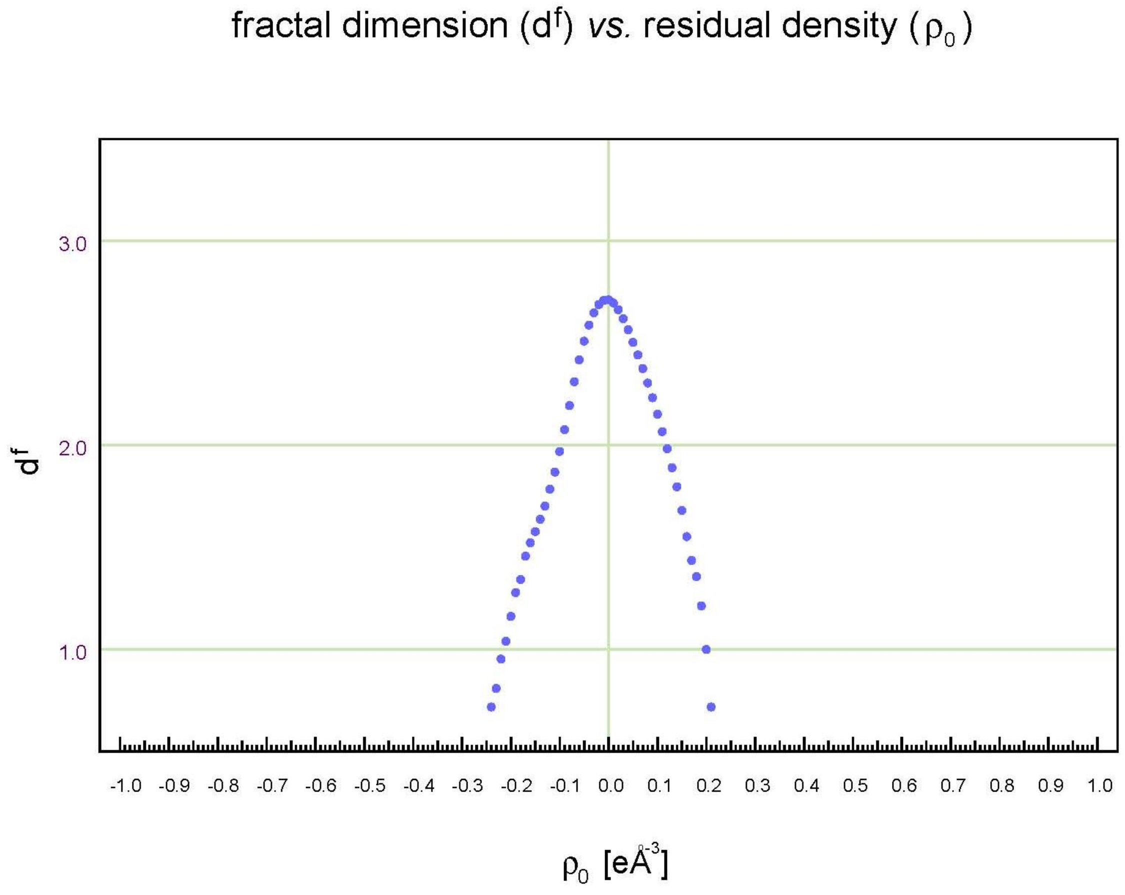


```
MODEL *model 4 4 0 0

FOUR     fmod1 4 4 0 0  fmod2 -1 2 0 0

SELECT   *fobs *fmod1 fmod2 print snlmin 0. snlmax 2.
GRID     3-points perp *cryst
ATOM     label S(1) symm 1 trans 0 0 0 *mark on plot
ATOM     label C(3) symm 1 trans 0 0 0 *mark on plot
ATOM     label O(4) symm 1 trans 0 0 0 *mark on plot
LIMITS   xmin  0.0 xmax 1.0 nx 70
LIMITS   ymin  0.0 ymax 1.0 ny 60
LIMITS   zmin  0.0 zmax 1.0 nz 50
```

```
d^f(0) = 2.7118
ρmin(d=2) = -0.0971 eA^-3
ρmax(d=2) = 0.1180 eA^-3

nx=70        rho_min: -0.24 eA^-3
ny=60        rho_max: 0.22 eA^-3
nz=50        delta rho: 0.46 eA^-3
```

Figure S3 Plot of the fractal dimension d^f vs. the residual electron density (ρ_0) in the unit cell of **Bic**. No resolution cutoff was applied to the data used for the Fourier transformation.

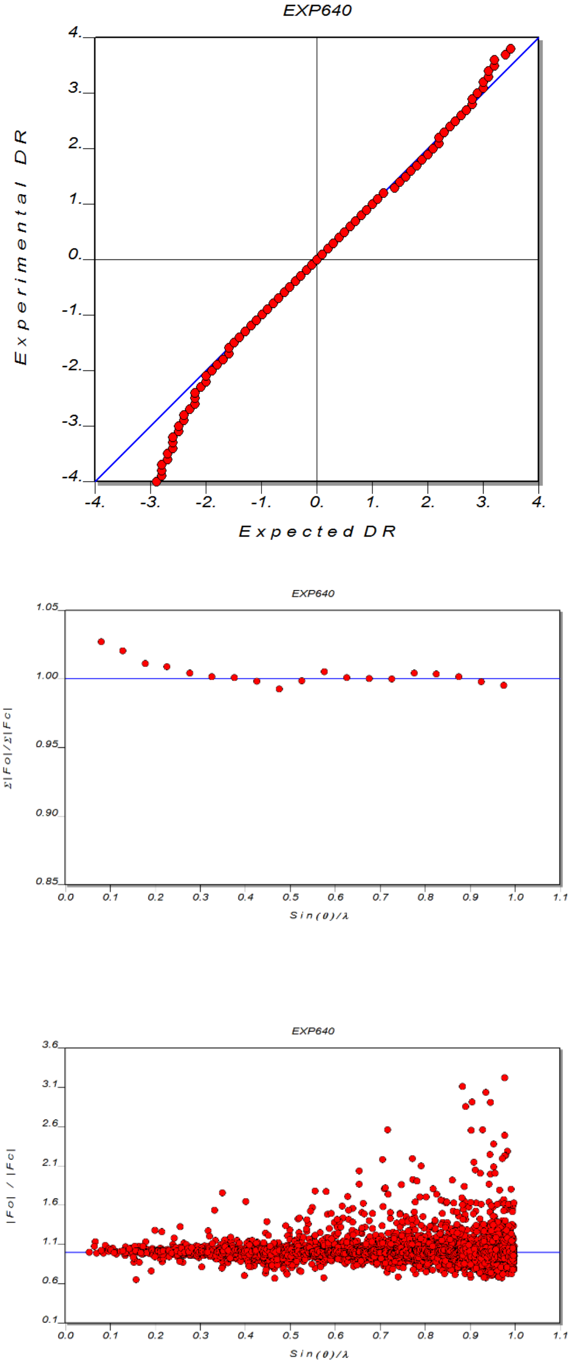

(a)

(b)

(c)

Figure S4 (a) Normal probability plot plotted against full dataset of **Bic**; (b) Scale factor plot against resolution; (c) $|F_o|$ / $|F_c|$ against resolution.

Charge Density Properties

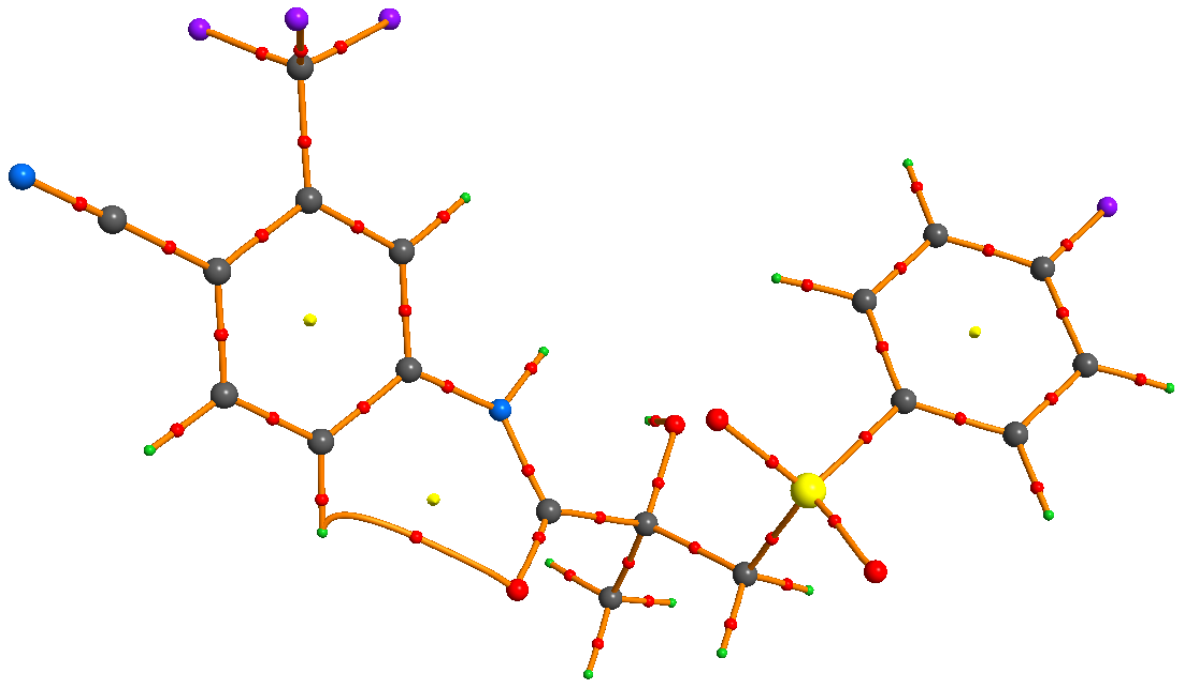

Figure S5 Molecular graph of **Bic**. Red and yellow circles denote CP (3,-1) and (3,+1).

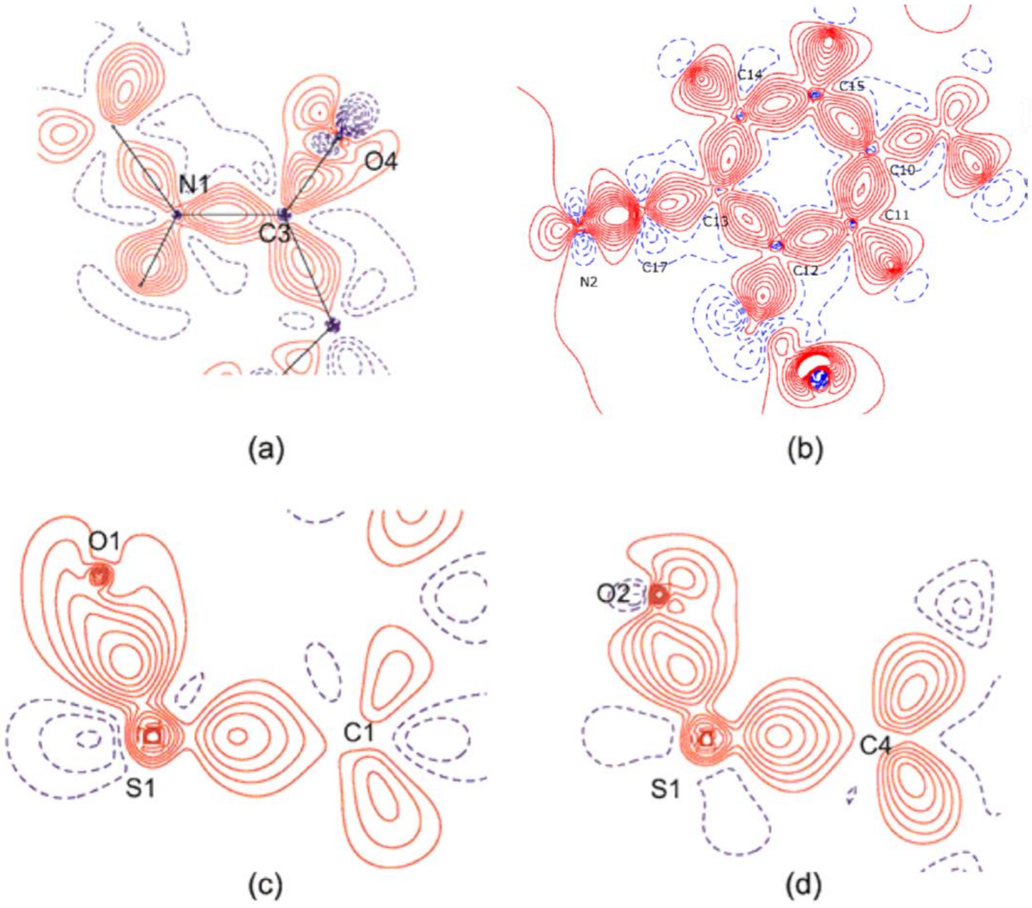

Figure S6 Experimental static deformation density in **Bic** depicted in the sections of (a) N(1), C(3), O(4); (b) N(2), C(11), C(15); (c) S(1), O(1), C(1); (d) S(1) O(2), C(4) atoms. The contour levels are equal to ±0.1, ±0.2, ±0.3, ±0.4, ±0.5, ±0.6, ±0.7, ±0.8, ±0.9, ±1.0 e Å^{-3}; the positive contours are shown with solid red line, the negative contours are dashed blue.

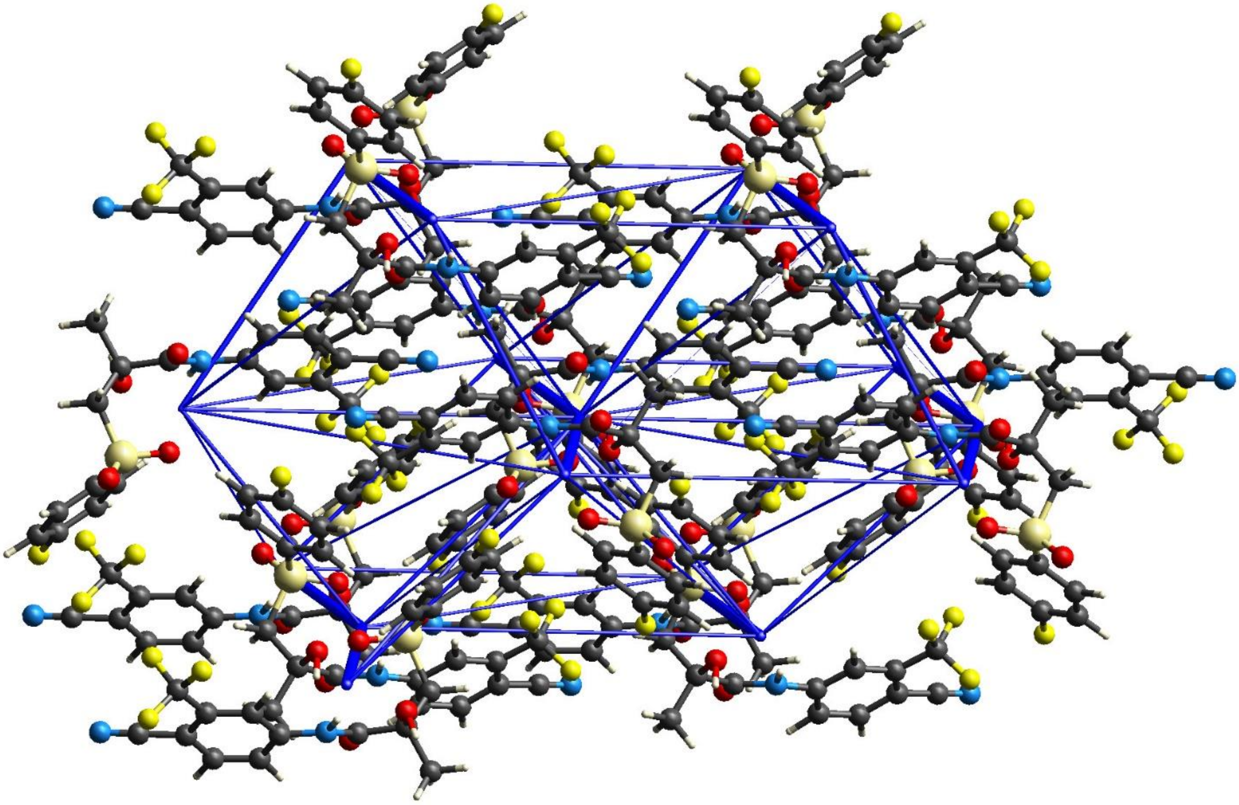

Figure S7 Energy framework for crystal packing of **Bic**. The width of blue line is related to the strength of intermolecular binding (E_{tot}).

Table S1 Characteristics of intramolecular CPs of **Bic** [a.u.]

Type CP	Bond	ρ(r)	$\nabla^2\rho(r)$	g(r)	v(r)	h(r)
(3,-1)	S(1)-O(1)	0.382	-0.313	0.524	-1.127	-0.602
(3,-1)	S(1)-O(2)	0.359	-0.314	0.469	-1.016	-0.547
(3,-1)	S(1)-C(1)	0.212	-0.292	0.167	-0.407	-0.240
(3,-1)	S(1)-C(4)	0.231	-0.453	0.175	-0.463	-0.288
(3,-1)	F(1)-C(8)	0.283	-0.737	0.227	-0.638	-0.411
(3,-1)	F(2)-C(16)	0.310	-0.676	0.296	-0.760	-0.465
(3,-1)	F(3)-C(16)	0.334	-1.767	0.167	-0.776	-0.609
(3,-1)	F(4)-C(16)	0.320	-1.784	0.132	-0.711	-0.578
(3,-1)	O(3)-C(18)	0.291	-1.022	0.196	-0.647	-0.451
(3,+1)		0.022	0.138	0.028	-0.021	0.007
(3,-1)	O(3)-H(3)	0.325	-1.227	0.238	-0.782	-0.544
(3,-1)	O(3)-H(1)	0.025	0.106	0.024	-0.021	0.003
(3,-1)	O(4)-C(3)	0.422	-1.384	0.452	-1.250	-0.798
(3,-1)	O(4)-H(15)	0.015	0.058	0.012	-0.010	0.002
(3,-1)	N(1)-C(3)	0.333	-1.118	0.272	-0.823	-0.551
(3,-1)	N(1)-C(11)	0.305	-0.888	0.250	-0.721	-0.472
(3,+1)		0.012	0.061	0.012	-0.009	0.003
(3,-1)	N(1)-H(1)	0.331	-1.275	0.243	-0.805	-0.562
(3,-1)	N(2)-C(17)	0.512	-1.457	0.699	-1.761	-1.063
(3,-1)	C(1)-C(18)	0.250	-0.549	0.193	-0.522	-0.330
(3,-1)	C(1)-H(1B)	0.281	-0.804	0.213	-0.627	-0.414
(3,-1)	C(1)-H(1A)	0.294	-0.855	0.230	-0.674	-0.444
(3,-1)	C(2)-C(3)	0.257	-0.656	0.189	-0.542	-0.353
(3,-1)	C(2)-C(18)	0.260	-0.649	0.197	-0.556	-0.359
(3,-1)	C(4)-C(5)	0.307	-0.672	0.290	-0.748	-0.458
(3,+1)		0.021	0.139	0.028	-0.021	0.007
(3,-1)	C(4)-C(9)	0.325	-0.936	0.285	-0.804	-0.519
(3,-1)	C(5)-C(6)	0.315	-0.796	0.285	-0.770	-0.484
(3,-1)	C(5)-H(5)	0.289	-0.840	0.224	-0.657	-0.434
(3,-1)	C(6)-C(7)	0.331	-0.961	0.294	-0.829	-0.534
(3,-1)	C(6)-H(6)	0.298	-0.948	0.224	-0.684	-0.460
(3,-1)	C(7)-C(8)	0.339	-1.079	0.294	-0.858	-0.564
(3,-1)	C(8)-C(9)	0.330	-0.892	0.305	-0.833	-0.528
(3,-1)	C(8)-H(8)	0.289	-0.830	0.225	-0.658	-0.433

(3,-1)	C(9)-H(9)	0.285	-0.818	0.219	-0.642	-0.423
(3,-1)	C(10)-C(11)	0.324	-0.932	0.284	-0.800	-0.517
(3,+1)		0.022	0.127	0.026	-0.021	0.006
(3,-1)	C(10)-C(15)	0.320	-0.889	0.281	-0.785	-0.504
(3,-1)	C(11)-C(16)	0.326	-0.963	0.284	-0.809	-0.525
(3,-1)	C(11)-H(11)	0.283	-0.801	0.217	-0.634	-0.417
(3,-1)	C(12)-C(17)	0.309	-0.833	0.266	-0.741	-0.475
(3,-1)	C(12)-C(16)	0.285	-0.844	0.214	-0.638	-0.425
(3,-1)	C(14)-C(17)	0.321	-0.839	0.291	-0.792	-0.501
(3,-1)	C(13)-C(17)	0.292	-0.800	0.235	-0.670	-0.435
(3,-1)	C(14)-C(15)	0.323	-0.940	0.281	-0.797	-0.516
(3,-1)	C(14)-H(14)	0.291	-0.906	0.216	-0.659	-0.443
(3,-1)	C(15)-H(15)	0.293	-0.905	0.221	-0.668	-0.447
(3,-1)	C(18)-H(18A)	0.272	-0.615	0.225	-0.604	-0.379
(3,-1)	C(18)-H(18B)	0.254	-0.623	0.189	-0.533	-0.344
(3,-1)	C(18)-H(18C)	0.260	-0.579	0.207	-0.560	-0.352

Table S2 The QTAIM integrated parameters for **Bic**.

Atom	Q_{exp}	V_{AIM}	Atom	Q_{exp}	V_{AIM}
**S1**	1.65	8.21	**C12**	0.32	9.70
**F1**	-0.64	17.65	**C13**	0.21	11.39
**F2**	-0.81	17.22	**C14**	0.29	11.02
**F3**	-0.66	17.35	**C15**	0.27	10.52
**F4**	-0.75	16.42	**C16**	1.64	3.54
**N1**	-2.37	16.07	**C17**	1.01	10.89
**N2**	-2.30	18.31	**C18**	0.11	9.00
**O1**	-1.05	14.76	**H3**	0.55	2.56
**O2**	-1.21	18.13	**H1**	0.47	2.80
**O3**	-0.23	14.72	**H1B**	-0.09	7.78
**O4**	-0.11	22.00	**H1A**	-0.08	6.91
**C1**	0.05	7.58	**H5**	-0.09	6.54
**C2**	0.76	5.06	**H6**	-0.11	9.20
**C3**	1.59	4.82	**H8**	-0.07	6.87
**C4**	-0.05	11.37	**H9**	-0.03	8.79
**C5**	0.31	10.88	**H11**	-0.04	6.70
**C6**	0.13	11.25	**H14**	-0.10	8.73
**C7**	0.90	7.50	**H15**	-0.07	6.70
**C8**	-0.14	11.88	**H18A**	0.00	6.49
**C9**	0.16	12.19	**H18B**	-0.02	7.48
**C10**	0.45	9.97	**H18C**	-0.01	8.01
**C11**	0.18	10.48			

Table S3 Parameters of intermolecular contacts in **Bic**

Atom1	Atom2	R, Å	ρ(r), a.u.	$\nabla^2\rho(r)$, a.u.	V(r) , a.u.	E_{bond}, kJ/mol
F1	F4	3.061	0.003	0.017	-0.002	-2.4
	O3	3.275	0.003	0.015	-0.002	-2.1
F2	H6	2.432	0.006	0.032	-0.004	-5.2
	F3	3.334	0.002	0.009	-0.001	-1.1
F3	C6	3.311	0.003	0.011	-0.001	-1.7
	H1b	2.628	0.003	0.015	-0.002	-2.1
	H9	3.247	0.001	0.005	0.000	-0.6
F4	H1b	2.783	0.002	0.011	-0.001	-1.5
	H18c	3.061	0.003	0.014	-0.002	-2.0
	C13	3.285	0.004	0.016	-0.002	-2.6
O1	H8	2.255	0.010	0.034	-0.008	-10.0
O2	H5	2.479	0.006	0.025	-0.003	-4.1
	H11	2.402	0.006	0.030	-0.004	-4.7
	H8	2.953	0.003	0.012	-0.001	-1.8
	H9	2.871	0.003	0.014	-0.002	-2.1
O4	H3	2.223	0.012	0.049	-0.008	-10.5
	H18a	2.58	0.006	0.025	-0.003	-4.3
	H14	2.554	0.004	0.022	-0.002	-3.1
N2	H15	2.418	0.008	0.036	-0.005	-6.3
	H18a	2.665	0.008	0.027	-0.004	-5.4
	H1a	2.981	0.009	0.031	-0.005	-6.0
	H18b	2.962	0.004	0.015	-0.002	-2.3
	C11	3.416	0.004	0.014	-0.002	-2.5
C7	C6	3.392	0.005	0.015	-0.002	-2.6
C8	H11	3.031	0.004	0.013	-0.002	-2.3
C15	C15	3.488	0.004	0.011	-0.001	-1.8
	H18a	3.129	0.004	0.013	-0.002	-2.2
H5	H8	2.413	0.004	0.014	-0.002	-2.2
H9	H9	2.853	0.002	0.007	-0.001	-0.9
H14	H18b	2.485	0.002	0.009	-0.001	-1.2
H18b	H18b	2.992	0.005	0.019	-0.002	-3.0

* Contacts are found and verified using WinXPRO program. The environment of **1** was generated as cluster and contacts inside this cluster were analyzed.

Bicalutamide in the binding pocket

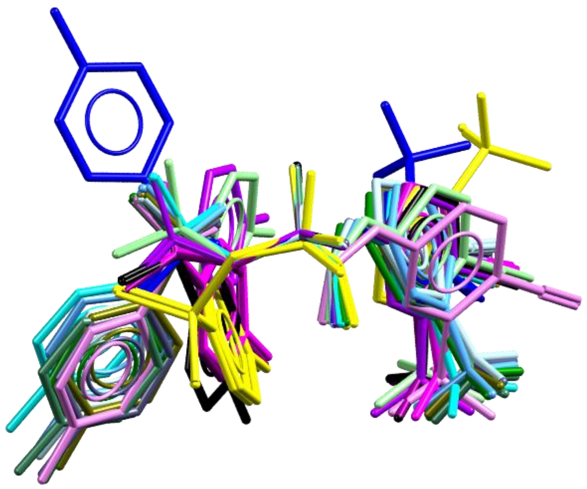

Figure S8 Molecular view of bicalutamide conformations in two polymorphs (monoclinic, blue; triclinic, black), four co-crystals (pink, magenta, violet, and light-bourdon) and ten complexes with macromolecule (yellow, light-blue and green colours). Hydrogen atoms are omitted. Superimposed atoms are the chiral carbon atoms and their four neighbours.

Table S4 Table 4. Electrostatic interaction energy (kJ mol^{-1}) between **Bic** and residues of LBP of the human serum albumin calculated by the EP/MM method. Selected complex is 4okl (2.4Å). A and B description indicate the chain name.

		human serum albumin	
		4okl_A	4okl_B
LEU	115	-0.1	0.5
VAL	116	0.0	0.0
ARG	117	5.4	5.2
PRO	118	0.6	0.6
VAL	122	0.0	0.0
MET	123	-0.1	0.0
ALA	126	-0.7	-0.7
THR	133	0.0	0.0
PHE	134	0.2	0.2
LEU	135	-0.2	-0.2
LYS	137	-4.7	-4.5
TYR	138	0.3	0.3
GLU	141	1.9	1.7
TYR	161	-0.2	-0.2
PHE	165	0.0	0.1
LEU	182	-0.2	-0.1
ARG	186	-1.3	-1.3
Σ		0.9	1.4

References

[S1] Meindl, K; Herbst-Irmer, R.; Henn, J. (2010). *Acta Crystallogr*. A**66**, 362-371.

[S2] Meindl, K; Henn, J. (2008). *Acta Crystallogr.* A**64**, 404-418.

[S3] Hubschle, C. B.; Dittrich, B. (2011). *J. Appl. Crystallogr.* **44**, 238−240.

[S4] Zhurova, E.A.; Zhurov, V.V.; Kumaradhas, P.; Cenedese, P.; Pinkerton, A.A.; J. Phys. Chem. B., **34**, 120, 8882-8891

[S5] Zhurov, V. V.; Zhurova, E. A. & Pinkerton, A. A. (2008). *J. Appl. Crystallogr.* **41**, 340-349.

[S6] Henn, J. & Meindl, K. (2014). *Acta Crystallogr*. A**70**, 248-256.
